# Zika virus infection in pregnant rhesus macaques causes placental dysfunction and immunopathology

**DOI:** 10.1038/s41467-017-02499-9

**Published:** 2018-01-17

**Authors:** Alec J. Hirsch, Victoria H. J. Roberts, Peta L. Grigsby, Nicole Haese, Matthias C. Schabel, Xiaojie Wang, Jamie O. Lo, Zheng Liu, Christopher D. Kroenke, Jessica L. Smith, Meredith Kelleher, Rebecca Broeckel, Craig N. Kreklywich, Christopher J. Parkins, Michael Denton, Patricia Smith, Victor DeFilippis, William Messer, Jay A. Nelson, Jon D. Hennebold, Marjorie Grafe, Lois Colgin, Anne Lewis, Rebecca Ducore, Tonya Swanson, Alfred W. Legasse, Michael K. Axthelm, Rhonda MacAllister, Ashlee V. Moses, Terry K. Morgan, Antonio E. Frias, Daniel N. Streblow

**Affiliations:** 1 The Vaccine & Gene Institute, Oregon Health and Science University (OHSU), 505 NW 185th Ave, Beaverton, 97006 USA; 2 Division of Pathobiology & Immunology, Oregon National Primate Research Center (ONPRC), 505 NW 185th Ave, Beaverton, 97006 USA; 3 Division of Reproductive & Developmental Sciences, ONPRC, 505 NW 185th Ave, Beaverton, 97006 USA; 4 Department of Obstetrics & Gynecology, OHSU, 3181 Sam Jackson Park Road, Portland, OR 97239 USA; 5 Advanced Imaging Research Center, OHSU, 3181 Sam Jackson Park Road, Portland, OR 97239 USA; 6 Utah Center for Advanced Imaging Research, Department of Radiology, University of Utah, 201 President’s Circle, Salt Lake City, UT 84112 USA; 7 Department of Molecular Microbiology & Immunology, OHSU, 3181 Sam Jackson Park Road, Portland, OR 97239 USA; 8 Department of Medicine, Division of Infectious Diseases, OHSU, 3181 Sam Jackson Park Road, Portland, OR 97239 USA; 9 Neuropathology, OHSU, 3181 Sam Jackson Park Road, Portland, OR 97239 USA; 10 Pathology Services Unit, Division of Comparative Medicine, ONPRC, 505 NW 185th Ave, Beaverton, 97006 USA; 11 Clinical Services Unit, Division of Comparative Medicine, ONPRC, 505 NW 185th Ave, Beaverton, 97006 USA; 12 Department of Pathology, OHSU, 3181 Sam Jackson Park Road, Portland, OR 97239 USA

## Abstract

Zika virus (ZIKV) infection during pregnancy leads to an increased risk of fetal growth restriction and fetal central nervous system malformations, which are outcomes broadly referred to as the Congenital Zika Syndrome (CZS). Here we infect pregnant rhesus macaques and investigate the impact of persistent ZIKV infection on uteroplacental pathology, blood flow, and fetal growth and development. Despite seemingly normal fetal growth and persistent fetal-placenta-maternal infection, advanced non-invasive in vivo imaging studies reveal dramatic effects on placental oxygen reserve accompanied by significantly decreased oxygen permeability of the placental villi. The observation of abnormal oxygen transport within the placenta appears to be a consequence of uterine vasculitis and placental villous damage in ZIKV cases. In addition, we demonstrate a robust maternal-placental-fetal inflammatory response following ZIKV infection. This animal model reveals a potential relationship between ZIKV infection and uteroplacental pathology that appears to affect oxygen delivery to the fetus during development.

## Abstract (web summary)

Zika virus infection during pregnancy can result in birth defects, but underlying pathogenesis at the maternal-fetal interface is unclear. Here, the authors use non-invasive in vivo imaging of Zika-infected rhesus macaques and show that infection results in abnormal oxygen transport across the placenta.

## Introduction

In general, both transplacental passage of maternal viral infections and subsequent fetal infection are rare^1^. When maternal viral infection does occur, it leads to productive viral replication in the placenta and a fetal inflammatory response that can have deleterious outcomes, even though the virus is not always detected in the fetus^1^. Zika virus (ZIKV), a mosquito-borne flavivirus closely related to yellow fever and dengue viruses, recently caused an epidemic in the Americas^2^. Historically, ZIKV infections have been sporadic and associated with relatively mild disease. However, clinical observations of the recent epidemic indicate that ZIKV may cause neurological sequelae in adults, such as Guillain–Barré Syndrome as well as a spectrum of neurological birth defects in infected fetuses, including microcephaly, eye defects and hearing loss^3,4^. While initial reports from the WHO and CDC highlighted microcephaly as the major concern with vertical transmission of ZIKV infection in pregnancy, newer studies refer to Congenital Zika Syndrome (CZS), of which microcephaly is one severe manifestation of infection. The data from a cohort of >1000 pregnant women with possible ZIKV infection indicated that ~5% of the resultant neonates had evidence of birth defects, and this proportion was ~10% if restricted to cases with confirmed evidence of ZIKV infection^5^. Within the spectrum of anomalies of CZS, there are a range of adverse obstetric outcomes, which occur in the absence of severe neonatal CNS deficits^6,7^. Viral infections during pregnancy have been demonstrated to cause spontaneous abortions, stillbirth, fetal infection, intrauterine growth restriction (IUGR), oligohydramnios, preterm premature rupture of membranes and preterm delivery; such outcomes have been strongly associated with ZIKV infection during pregnancy^8,9^. However, infection with other flaviviruses such as dengue virus are rarely associated with fetal infection or severe birth defects^10^, suggesting that ZIKV is quite unique in this regard. Furthermore, these observations strongly suggest a component of placental dysfunction in ZIKV cases, which has not previously been extensively investigated in vivo.

Prompted by the ZIKV epidemic, recent efforts to understand the detrimental effects of this virus on the developing fetus and pregnancy outcomes have expanded exponentially. The presence of ZIKV RNA in the mother, fetal brain and amniotic fluid (AF) during human pregnancy has been reported^11–13^. Much of the attention to date has been focused on the biology of ZIKV^14^, the *Aedes spp*. mosquito vector control^15^, birth defects including microcephaly^16–18^, and sexual transmission of the virus^19,20^. However, because of the immunologic complexity of pregnancy, the maternal-feto-placental immune responses and neonatal neurodevelopmental consequences from ZIKV infection in utero have yet to be fully explored. Recent studies by our group and others^21–26^ identified the presence of ZIKV RNA in the plasma of experimentally infected rhesus monkeys, as well as a robust immune response, including ZIKV-specific antibodies and T-cells in non-pregnant adult animals. ZIKV infection of a single pregnant pigtail macaque (*Macaca nemestrina)* resulted in several sequelae in the fetus reminiscent of CZS in humans, including restricted fetal brain growth and the presence of viral RNA in the fetal brain^27^. Additionally, infection of pregnant rhesus macaques using the French Polynesian ZIKV strain has similarly demonstrated some evidence of disrupted fetal growth, prolonged maternal viremia, and inflammation at the maternal-fetal interface, including mild decidual perivascular inflammation (not unusual in human decidua) and placental acute chorioamnionitis, which is almost always associated with a bacterial intra-amniotic infection^28^. However, the impact of ZIKV infection on in vivo uteroplacental blood flow and oxygen transport has not previously been examined or correlated with in vitro placental histology.

As the placenta has a critical role in fetal development and pregnancy success, attention has been focused on the mechanisms by which ZIKV transmission occurs, the placental cell types impacted and the adverse effects of maternal ZIKV infection^29–35^. However, placenta studies are impeded by the inability to examine placental function in vivo, with most data generated from post-delivery in vitro studies. Recently, we have made advancements to overcome this hurdle through the development of placenta-specific magnetic resonance imaging (MRI) protocols that provide quantitative assessment of placental perfusion and oxygenation in vivo. These protocols have been developed and validated in nonhuman primate models of perturbation during pregnancy^36,37^. Like humans, the nonhuman primate has a long gestation and a hemochorial placenta, making this a well-suited translational model to understand human disease mechanisms. The available data from nonhuman primate studies and human cohorts suggest some underlying inflammation contributing to the placental dysfunction in ZIKV-infected pregnancies, but prior to now, such assessments have not been linked to an in vivo functional correlate.

In order to investigate the effect of maternal ZIKV infection during pregnancy, five pregnant rhesus macaques were infected with ZIKV at different time points across gestation. We hypothesized that ZIKV infection would induce both placental and fetal inflammation and perturb placental function. Maternal viral loads as well as innate and adaptive immune responses were measured longitudinally following ZIKV infection. Chronological evaluation of fetal growth, uteroplacental blood flow and amniotic fluid index were assessed via non-invasive ultrasound (US) throughout pregnancy. Moreover, we employed our in vivo imaging capabilities to quantify placental perfusion and oxygenation following maternal ZIKV infection. Uteroplacental pathology was reviewed to test for correlations between tissue structure and functional assay results. Comprehensive immunophenotyping and quantification of viral RNA in maternal, placental and fetal tissue samples was performed post-delivery.

## Results

### Nonhuman primate model of ZIKV during pregnancy

**Fig. 1:**
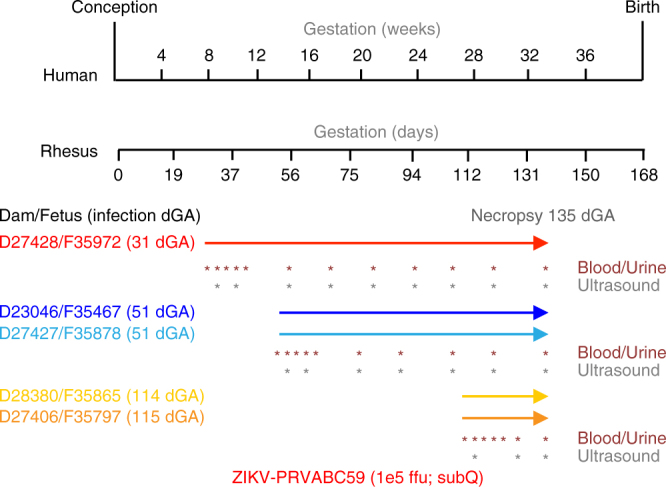
Experimental design and ZIKV infection relative to fetal brain development. The pregnant dams were infected with ZIKV at 31, 51, 114, and 115 dGA. For all animals, C-section delivery was performed at 135dGA and maternal and fetal tissues were collected. Maternal peripheral blood, urine, and saliva samples (red asterisks) were obtained at the times indicated. Ultrasound analyses of the fetus and placenta were performed at the times indicated with gray asterisks

Five pregnant rhesus macaques (time-mated breeding) were infected with ZIKV (strain PRVABC59) at 31 days of gestation (dGA) (animal ID: D27428), 51 dGA (animals: D23046 and D27427), 114 dGA (animal: D28380) and 115 dGA (animal: D27406). (Throughout this manuscript, animal identification numbers will be preceded by “D”, indicating dams or “F” indicating fetuses). Full term in rhesus macaques is 168 days. The dams received ZIKV inoculum (1 × 10^5^ ffu) divided over 10 subcutaneous injections in the hand, wrist and upper arm (1 × 10^4^ ffu per injection site)^22^. Each pregnancy was monitored following infection by serial physical exams, blood counts, a total chemistry panel and ultrasound. Cesarean section delivery was performed for all animals at 135dGA, at which time maternal and fetal tissues were collected and processed (Fig. 1). Six uninfected control rhesus macaques from two ongoing studies were used as a comparative data set for all placental parameters unless otherwise noted. These animals underwent advanced imaging, were similarly delivered at 135dGA, and experienced no treatments or manipulations during pregnancy.

### Innate and adaptive immune responses after ZIKV infection

We monitored the development of the maternal immune response post-ZIKV infection by utilizing PBMCs isolated from blood samples at various days post infection (dpi). PBMCs were phenotypically analyzed by flow cytometry using antibody panels designed to identify innate immune cell activation as well as adaptive immune T- and B-cell proliferative responses. Innate immune cells were classified as monocyte/ macrophages, myeloid dendritic cells (DC), natural killer (NK) cells, or other (non-myeloid DC) based on the gating strategy described in “Methods” section. Within 5 dpi, all dams showed innate immune cell activation as demonstrated by the presence of CD169^+^ staining within monocyte/ macrophage, myeloid DC, and NK subsets (Supplementary Fig. 1a–c). Activation of NK cells was observed from 12–21 dpi in some dams, additionally, DCs and NK cells displayed elevated activation at 70 and 85 dpi. CD8 T-cell proliferation peaked from 5 to 14 dpi for naive, effector memory (T_EM_), and central memory (T_CM_) subsets with a second time point of proliferation for the T_EM_ and T_CM_ subsets at 56 dpi. B-cell proliferative burst responses were maximal at 10–14 dpi (Supplementary Fig. 1d–f). ZIKV-specific maternal antibodies were detected as early as 6 dpi (Supplementary Fig. 2), and antibody titers increased through 28 dpi. Analysis of maternal and fetal serum collected at 85 dpi showed neutralizing activity (Supplementary Fig. 2).

### Maternal viral loads and tissue distribution

During the period between infection and delivery of the fetus, viral RNA was detected in the dam’s serum from four out of five animals at 3 dpi, which is consistent with previous reports of ZIKV infection in rhesus macaques^21,22^. Serum from animal D23046 was not sampled between days 1 and 6 post infection and was negative for viral RNA at 7 dpi. However, viral RNA was detected in the urine for this animal for most of the time points (2–85 dpi, Supplementary Fig. 3). In animal D27428 (31 dGA), viral RNA in the urine was detected sporadically through 84 dpi; whereas the other animals had more limited detection profiles. A number of maternal tissues were positive for ZIKV RNA at necropsy, including the genitourinary tract, brain, peripheral nerves and spinal cord, as well as joint and muscle tissues (Supplementary Table 1), demonstrating that ZIKV persists in maternal tissues and is shed in urine for extended periods of time post infection.

### Fetal viral loads and tissue distribution

Viral RNA was detected in the urine of all five fetuses, indicating that all were infected and persistently shedding viral RNA in their urine (Supplementary Table 2). In addition, RNA was detected in tissue from all of the fetuses except animal F35878 (51 dGA infection) (Supplementary Table 3). Viral RNA-positive tissues included spinal cord, trigeminal cranial nerve, brachial plexus, peripheral nerves, eye, thyroid, pituitary gland, thymus, tonsil, finger joints, elbow, hamstring, bladder, uterus, myometrium, cervix, and ovary (Supplementary Table 3). In addition, amniotic fluid of fetus F35467 was positive for viral RNA at necropsy (85 dpi), and animals F35972 (104 dpi) and F35878 (85 dpi) had detectable levels of viral RNA in their CSF (Supplementary Table 2). The brain of animal F35797 was examined for viral RNA, following observation of anomalous fetal brain MRI (see below), and a detectable level of RNA was found. Combined, these data indicate that each of the fetuses were infected following subcutaneous infection of the dams and that viral RNA persisted; the latter is consistent with our previous findings in non-pregnant rhesus macaques^22^.

### Placental viral loads

The hemochorial placenta of the rhesus macaque is similar to that of the human, but is distinguished by two lobes^38^. The umbilical cord attaches centrally to a primary lobe and clusters of bridging vessels supply a structurally independent secondary lobe. To comprehensively assess viral load in the placenta, RNA was isolated from each individually mapped and identified cotyledon from both the primary and secondary lobes (Table 1). In humans and nonhuman primates, cotyledons are perfusion domains defined by septa, which include a maternal blood pool (intervillous space) and fetal vasculature within the villous tissue for nutrient exchange^38^. As shown in Table 1, ZIKV RNA was detected in placental tissues from four of the five animals (D23046, D27427, D27406 and D28380). For animal D23046, two sections were positive in the primary placenta: peripherally located cotyledons #3 and #5; and three in the secondary placenta: peripherally located cotyledon #1, #3 and #6 (Supplementary Fig. 7 shows gross photos of each of the five ZIKV placentas with individually identified, annotated and numbered cotyledons depicting the relative location of each sample site). These findings suggest that placental infection may be focal, rather than diffuse and widespread. The two other positive animals had reduced numbers of positive cotyledons (Table 1). While the exact route and timing of placental transfer and fetal infection relative to the dams is unknown, these data argue that ZIKV infects the placenta and fetal tissues, which can remain persistently viral RNA positive for 85 dpi.

**Table 1:** Detection of ZIKV RNA in placenta and maternal, placental and fetal characteristics

Individual perfusion domains (cotyledons) were dissected from primary and secondary placental lobes from ZIKV-infected RM and total RNA was prepared using the Trizol method. ZIKV RNA levels were quantified using qRT-PCR and presented in log10. Dash = not detected. Gray areas represent lack of perfusion domains. Maternal, fetal and placental weights, and the fetal:placental ratio in gestational age-matched control animals (*n* = 6, mean ± SD) and the ZIKV-infected animals (individually represented)

To confirm ZIKV infection of the placenta, trophoblast cells were isolated from placental tissue from three animals (D27428, D27427, and D28380). Cells were maintained in culture for 3 days and used for detection of ZIKV envelope (E) protein by immunofluoresence analysis (IFA) at 1, 2, and 3 days post-plating (dpp). Supernatants conditioned for 24 h were collected immediately prior to IFA and viral titers determined on Vero cells by plaque assay. Gradient-banded cells stained strongly for cytokeratin 7 (Supplementary Fig. 4a), while phase microscopy confirmed the presence of mononuclear cytotrophoblasts (CTB) at 1 dpp with fusion to yield differentiated syncytiotrophoblasts (STB) by 3 dpp (Supplementary Fig. 4b). While trophoblasts were the predominant cell type in these cultures, occasional cells with the morphology of Hofbauer cells (HC) and lymphocytes were observed. Infectious virus was recovered from the placental cultures derived from animal D28380 (0.33 pfu/ml on 1 dpp; 100 pfu/ml on 2 dpp; 100 pfu/ml on 3 dpp), but not from the other two animals. In cultures from all three animals however, cells expressing ZIKV E protein were occasionally observed. These were typically trophoblasts but cells resembling HC and lymphocytes were also found (Supplementary Fig. 4c). In keeping with infectious virus recovery, placental cultures derived from animal D28380 contained the highest number of ZIKV E-expressing cells, possibly reflecting the time elapsed between maternal infection and tissue collection (21 days).

### Placental monocyte and T-cell subsets and activation status

**Fig. 2:**
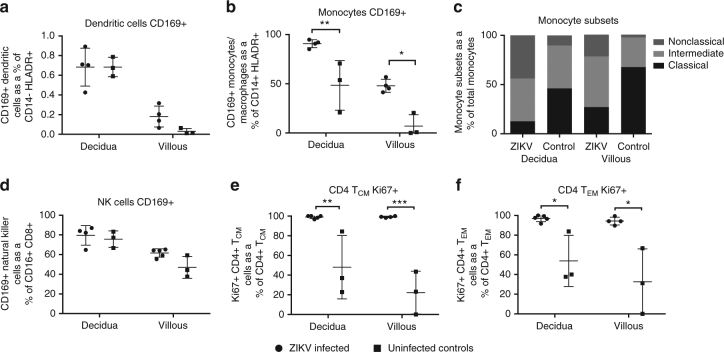
ZIKV infection alters decidua and villous placenta monocyte and CD4+ T-cell populations and activation status. Decidua and villous placental lymphocytes were isolated from ZIKV-infected and control RM and analyzed by flow cytometry for the presence of the cellular activation marker CD169 on **a** dendritic cells; **b** monocytes; **c** monocyte subsets; and **d** NK cells, or the marker for Ki67 proliferation marker on **e** CD4+ central memory T cells and **f** CD4+ effector memory T cells. Cells were stained with a panel of antibodies recognizing HLA-DR, CD14, CD11c, CD123, CD20, CD3, CD16, and CD169 or a secondary panel containing CD3, CD4, CD8β, CD95, CD28, and Ki67. One-way ANOVA was performed with Tukey’s multiple comparison test, bars represent mean and SEM, ***p < 0.001, **p < 0.01, *p < 0.05

The immune response in decidua and villous tissues from ZIKV-infected dams at 135dGA was compared to uninfected gestational age-matched control dams. Decidua and villous tissues were processed to single-cell suspensions that were stained with flow cytometric antibody panels specific for innate and T-cell subsets. The frequency of activated innate immune cells (monocytes/macrophages and DCs) was higher in placental tissues from ZIKV-infected dams vs. control dams (Fig. 2a, b, d). There was a significant increase in the percent of activated (CD169^+^) monocyte/macrophages from ZIKV-infected placenta tissues compared to uninfected controls in both the decidua (*p* < 0.01, one-way ANOVA) and villous (*p* < 0.05, one-way ANOVA). The monocyte population was further divided into three functionally different subsets based on CD14 and CD16 expression: classical (CD14^hi^CD16^−^), intermediate (CD14^hi^CD16^+^), and non-classical (CD14^lo^CD16^+^) monocytes. Surprisingly, the ratio of classical to non-classical monocytes was decreased in both decidua and villous from ZIKV-infected dams compared to uninfected controls (Fig. 2c). Classical monocytes are involved in wound healing, antigen presentation, and typically secrete IL-10, while non-classical monocytes actively patrol tissues following damage and secrete inflammatory cytokines (IFN-γ, TNF-α, IL-1β, and IL-12)^39^. Staining of placenta tissues for macrophage subtypes via CD68 (M1) and CD163 (M2) showed both populations were present, with an increase in M1 macrophages in the villous stroma during ZIKV infection (Supplementary Fig. 5). Proliferation of CD4 T cells was also increased in placenta tissues from ZIKV-infected dams compared to uninfected controls. These differences were most evident and significant in the CD4 T_EM_ (*p* < 0.05, one-way ANOVA) and CD4 T_CM_ (*p* < 0.01, one-way ANOVA) cell subsets (Fig. 2e, f). These results suggest an active inflammatory immune response is maintained in the placenta following ZIKV infection.

### Cytokine and chemokine production

**Fig. 3:**
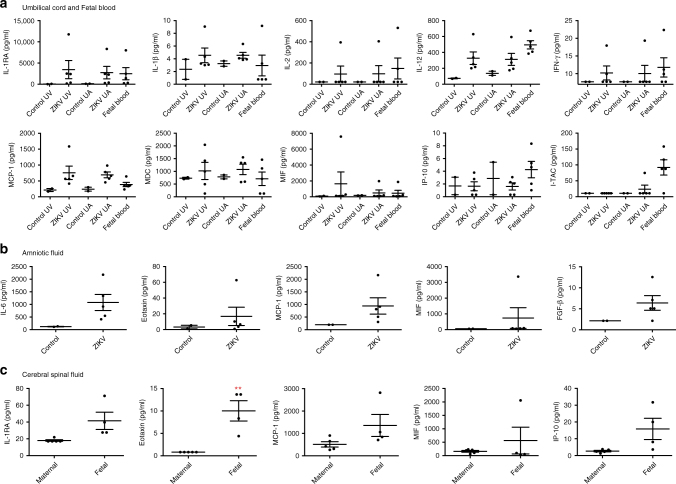
Cytokine and chemokine profiling. A 29-plex-cytokine magnetic bead assay was performed on **a** plasma isolated from ZIKV-infected and control fetal and cord blood, **b** amniotic fluid; and **c** fetal and maternal cerebral spinal fluid at 135dGA. Plasma analysis revealed changes in the cytokines IL-RA, IL-1β, IL-2, IL-12, IFNγ, and MIF; and chemokines MCP-1 (CCL-2), MDC (CCL-22), IP-10 (CXCL-10), and ITAC (CXCL-11). Compared to controls increased levels of IL-6, Eotaxin (CCL-11), MCP-1 (CCL-2), MIF and FGF-basic were detected in amniotic fluid from infected animals. Levels of IL-1RA, Eotaxin (CCL-11), MCP-1 (CCL-2), MIF, and IP-10 (CXCL-10) were higher in fetal vs. maternal CSF. One-way ANOVA was performed with Tukey’s multiple comparison test, bars represent mean and SEM, **p < 0.01, *p < 0.05 UV, umbilical vein; UC, umbilical cord

A Luminex NHP Cytokine Magnetic 29-plex panel was used to quantify cytokine and chemokine expression in umbilical blood, fetal circulation, and amniotic and cerebral spinal fluids. Fetal peripheral blood and arterial/venous umbilical cord blood were collected at delivery from ZIKV-infected animals (135dGA) and age-matched controls. Because of the direction of blood flow, inflammatory mediators present in fetal peripheral blood and umbilical artery can be considered to originate within the fetus, while inflammatory mediators present in the umbilical vein originate within the placenta. Similar expression of IL-1RA, IL-1β, IL-12, MCP-1 (CCL-2), MDC (CCL-22), IFNγ, and IP-10 (CXCL-10) was observed between fetal plasma, and the umbilical artery and vein circulations, implying that the placenta and fetus are immunologically responding to ZIKV (Fig. 3a). The production of inflammatory cytokines such as IFN-γ and IL-12 is consistent with the observed shift toward non-classical monocytes present in the placenta villous and decidua. However, the expression of I-TAC (CXCL-11) was predominately higher in fetal plasma. Comparatively, the expression of all these cytokines was minimal in the maternal circulation. Cytokine profiling of amniotic fluid revealed increased levels of IL-6, Eotaxin (CCL-11), MCP-1 (CCL-2), and FGF-β (Fig. 3b). Together these finding are quite dramatic and suggest that the fetus is undergoing a significant inflammatory immune response. Similarly, the CSF of the fetus had significantly elevated levels of IL-1RA, Eotaxin (CCL-11), MCP-1 (CCL-2) and IP-10 (CXCL-10) compared to the maternal CSF (*p* < 0.05, one-way ANOVA), which could be indicative of an ongoing viral infection (Fig. 3c).

### Assessment of uteroplacental blood flow

**Fig. 4:**
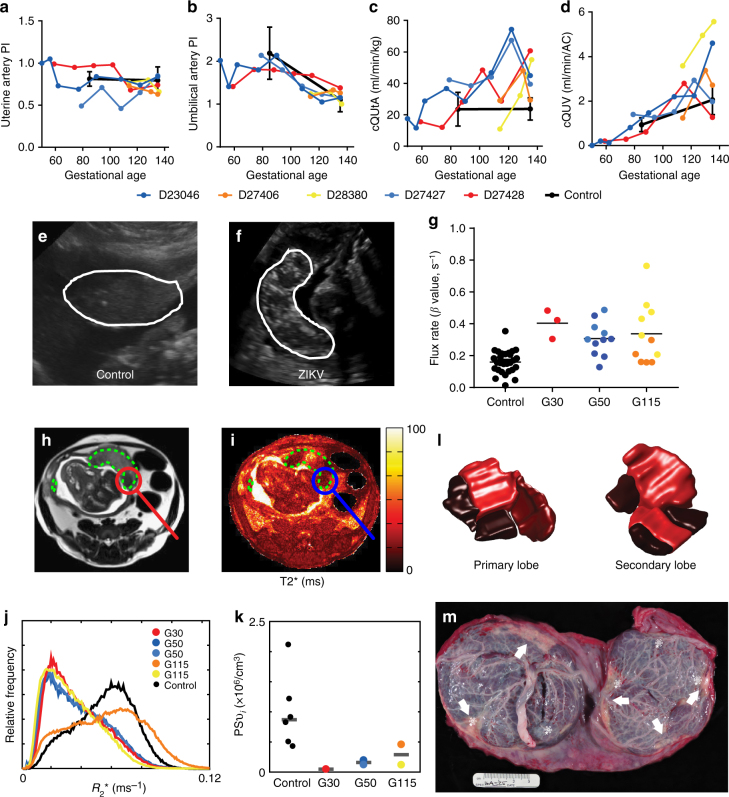
Ultrasound and MR imaging of utero-feto-placental blood flow. **a** Uterine artery pulsatility index, **b** uterine artery blood flow volume (cQUtA) corrected for maternal body weight, **c** umbilical artery pulsatility index, and **d** umbilical vein blood flow volume (cQUV) corrected for fetal abdominal circumference measured by Doppler ultrasound in the ZIKV-exposed animals and a cohort of control, non-infected rhesus macaques (*n* = 6, black closed circles; mean ± SEM) across gestation. **e** Ultrasound images showing the appearance of the placenta (white outline) in a representative control uninfected dam (102dGA) and **f** a ZIKV-infected dam at 61dpi (112dGA). Both images were acquired at the same gain setting. **g** CE-US calculated microvascular flux rate from control (*n* = 27 spiral artery sources from *n* = 7 animals) and ZIKV-infected animals. Co-registered axial MRI images from animal D23046 are shown in **h** (T2w HASTE acquisition) and **i** (quantitative *T*2* map). Regions of interest (ROIs) delineating both placental lobes are indicated by the dashed green lines, and a focal region of pronounced hypoperfusion is circled. **j** Plots normalized histograms of placental relaxation rate (*R*2* = 1/*T*2*) for all five ZIKV-infected animals (colored curves), along with the median histogram for the six control animals (black curve). **k** Plots median values of PSv_*i*_ (averaged over all placental lobules) for each of the six control animals (black points) versus values for the five ZIKV-infected animals (colored points), stratified by gestational age at time of infection. **l** Shows relative blood flow for both the primary and secondary placental lobes in D23046 determined from DCE-MRI measurements for the perfusion domains corresponding to each placental lobule, as determined from DCE-MRI measurements. Darker shades indicated regions of the placenta that are hypoperfused. **m** Image of the fetal side of the placenta in this animal, demonstrating infarcted regions (white arrows) corresponding to the regions of hypoperfusion seen on DCE-MRI. Of note, ZIKV RNA was detected at five sites across the two placental lobes, indicated by the white asterisk. These ZIKV + areas predominantly correspond to hypoperfused cotyledons. *p* < 0.0001 one-way ANOVA with Tukey’s multiple comparison test, ***p < 0.001, **p < 0.01

**Fig. 5:**
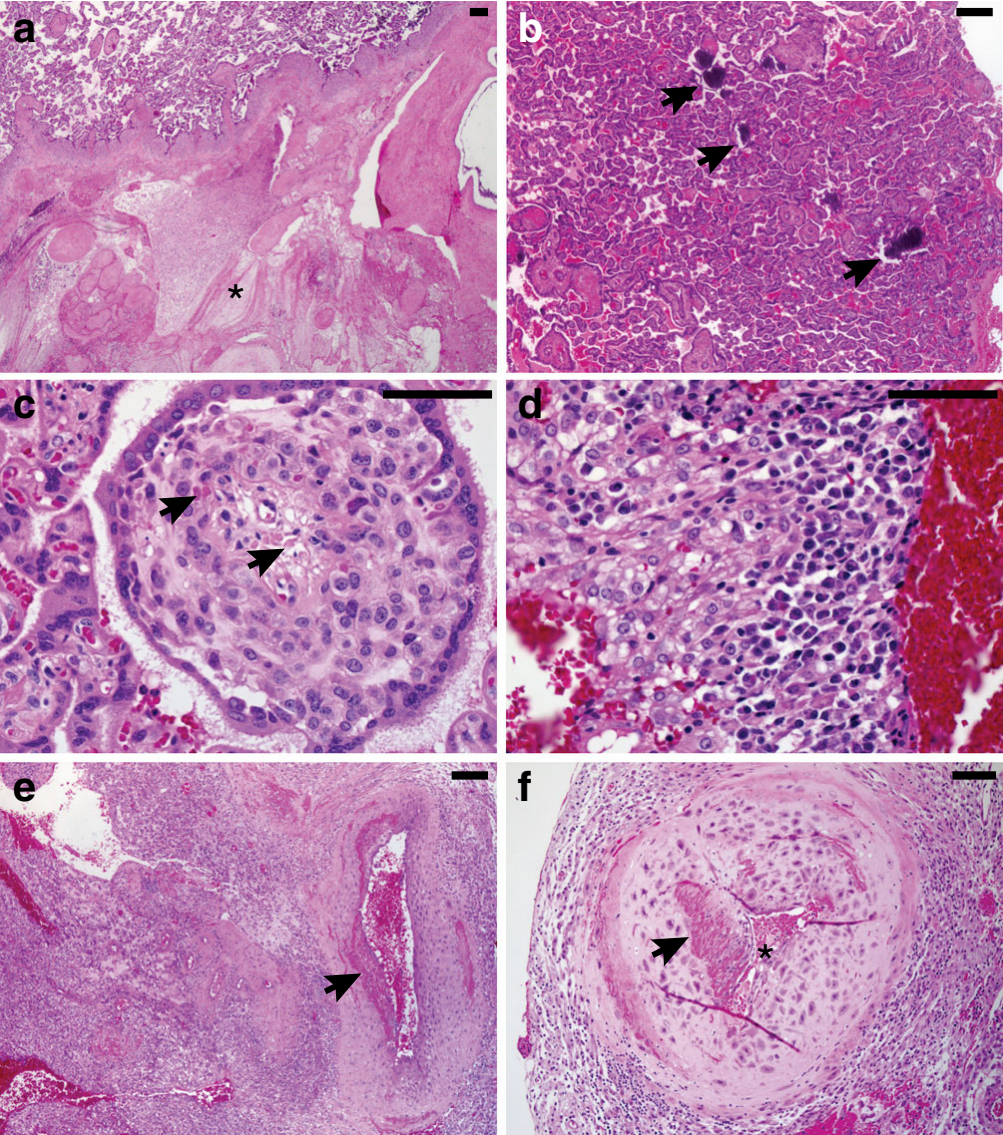
Uteroplacental histopathology of ZIKV-infected cases compared with gestational-age-matched negative controls. **a** All five ZIKV-infected cases showed placental infarctions (asterix) with large gross infarctions seen in the cases infected at days 31–51. **b** ZIKV cases had conspicuous villous stromal calcifications (arrows), which are a consequence of stromal fibroblast cell death (**c**). ZIKV cases were positive for chronic decidualitis with plasma cells and lymphoplasmacytic leuokocytoclastic vasculitis (**d**), which was associated damage to the uterine spiral arteries (**e**) and vascular luminal (astericks) narrowing (**f**). Scale bar is 100 µm

Longitudinal assessments of uteroplacental hemodynamics and fetal growth were made by ultrasound examinations prior to infection and across gestation until delivery. Neither the uterine artery pulsatility index (PI), nor the umbilical artery PI, deviated from control in any of the ZIKV animals (Fig. 4a, b). Control animal data were obtained at two gestational ages: 85dGA and 135dGA (Fig. 4a–d). Quantitative estimation of volumetric blood flow in the uterine artery with ultrasound has been used by our group and others to assess perfusion on the maternal side of the placenta^40–43^. The calculated blood flow volume in the uterine artery (cQuta) corrected for maternal body weight demonstrated some variability between ZIKV animals, but revealed an overall trend to increasing with advancing gestational age, as would be anticipated (Fig. 5c). Similarly, the quantitative estimation of blood flow on the fetal side of the placenta^41,44,45^, the calculated blood flow in the umbilical vein (cQuv) also increased across gestation, with values comparable to uninfected control animals, (Fig. 4d). While Doppler ultrasound provides a semi-quantitative estimation of blood flow, it is limited to measurements in major vessels both proximal and distal to the placental circulation contributing to a lack of sensitivity to detect placental dysfunction. Significantly, we observed an increase in echogenicity of the placenta in all three of the ZIKV animals infected early in gestation. This observation was consistently observed and all ultrasound exams were performed by one ultrasonographer (AEF) with similar image acquisition parameters. This finding was apparent at ~40 dpi (mid-gestation, Fig. 4f) and is suggestive of inflammation and/or ischemic injury. Given the increased echogenicity of the placenta and the limitations of Doppler ultrasound to assess placental function in vivo, we employed advanced non-invasive imaging modalities prior to delivery at 135dGA to further evaluate placental vascular function in vivo^36,37,46,47^.

Contrast-enhanced ultrasound (CEUS) with maternal infusion of Definity^®^ contrast agent was used to locate and visualize spiral artery sources supplying individual placental cotyledons from the ZIKV-infected animals and uninfected controls at 135dGA. The flux rate constant (*β*) provides a measure of microvascular resistance and gives an indirect measure of blood flow in the placental intervillous space^47^. In comparing microvascular flux rates from ZIKV-infected animals to data from uninfected control animals at 135dGA, we observed a significant increase in flux rate regardless of gestational age at the time of infection (Fig. 4g, *p* < 0.0001 one-way ANOVA). While our CEUS data analysis visualizes the spiral artery sources that supply individual placental cotyledons, it does not allow for absolute quantification of blood flow volumes, instead reflecting the change in vascular impedance. The increase in flux rate following in utero infection with ZIKV is indicative of increased velocity of maternal blood being delivered to the intervillous space from the spiral arteries, which may result in shear stress-induced placental injury.

### Placental perfusion and oxygenation by MRI

As CEUS may not reflect the global regional perfusion differences amongst cotyledons, we also employed novel MRI techniques for placental imaging at 135dGA^36,37^. Our group has recently developed MRI methods to quantify maternal perfusion of the placenta using both Dynamic Contrast-Enhanced MRI (DCE-MRI) to interrogate delivery and transport of maternal blood in the placenta^36,47^, and Blood Oxygen Level-Dependent (BOLD) MRI to characterize placental oxygenation, (via *T*2 * mapping)^37^. This technique enables us to separately quantify placental perfusion and placental oxygenation while accounting for the complex vascular network of the intervillous space. When comparing DCE-MRI results for the five ZIKV-infected animals to a group of six pregnant rhesus macaque controls matched at 135dGA, we found modest, but not statistically significant, decreases in both total placental blood flow (139 ml/min vs. 161 ml/min, respectively) and normalized placental blood flow (1.14 ml/ml/min vs. 1.25 ml/ml/min, respectively). Histograms of *R*2 * (= 1/*T*2*, normalized to placental volume) reveal that four of the five ZIKV-infected animals (excepting D27406) demonstrated a higher level of oxygenated maternal blood within the placenta as compared to the control group (Fig. 4j). We modeled the spatial distribution of *T*2* values within the placenta to estimate the ratio of oxygen permeability-surface area product to blood flow^37^. Combining these values with flow estimates from DCE-MRI, revealed large and statistically significant decreases (PSv_*i*_ = 1.22 × 10^6^ vs. PSv_*i*_ = 8.69 × 10^6^, *p* = 0.018) in oxygen permeability-surface area product in the ZIKV-infected dams relative to control animals. PSv_*i*_ is a parameter representing the efficiency of oxygen transport from intervillous space to the fetal villi and is quantitated as the product of fetal villous permeability, fetal villous surface area and intervillous space^37^. *P* values were calculated using a non-parametric two-sample Kolmogorov–Smirnov test. D27406 was also an outlier in these data, with oxygen permeability values in the low range of the control animals. Combined with our observation of nearly normal maternal blood flow and increased levels of oxygenated blood within the placenta, these results strongly suggest that ZIKV infection impairs transplacental transport of oxygen, potentially resulting in long-term fetal oxygen deprivation during gestation, likely secondary to placental damage occurring at the level of the fetal villi. Furthermore, high-resolution post-contrast imaging reveals both gross and scattered punctate regions of placental infarction with no significant contrast uptake (Fig. 4), indicating abnormal perfusion in the placenta from the ZIKV-infected dams. Lastly, comparison of the three-dimensional isosurfaces of the primary and secondary lobes generated by MRI analysis with the gross placenta post-delivery of one animal (D23046) showed close correspondence between areas of low perfusion and the infarcted regions (Fig. 4l, m). Of note, regions of low perfusion correspond to positive sites of ZIKV RNA detection (Fig. 4m, white asterisks).

### Placental and fetal growth after ZIKV exposure

Serial fetal biometry measurements made by ultrasound enabled fetal growth to be tracked across gestation. Abdominal circumference, head circumference, biparietal diameter, and femur length did not deviate from the reference data across gestation (Supplementary Fig. 6). Amniotic fluid levels were on the lower end of the normal control range at 135dGA for all ZIKV animals (Supplementary Fig. 6e). Fetal weight at 135dGA is reported in Table 1. Of note, there is variability across the five ZIKV-infected animals, but mean fetal weight did not differ from the control fetal cohort and we did not find evidence of fetal growth restriction following ZIKV infection. Importantly, cesarean section delivery of the fetus was performed at 135dGA, which corresponds to the early third trimester and the start of the exponential fetal growth period. Deviation from the normal growth trajectory may have manifested if the pregnancy had been continued closer to term of 168dGA. Placental weight was greater in the ZIKV-infected animals compared to controls; importantly, calculated fetal:placental weight ratio was decreased in 4 out of 5 animals. The fetal:placental ratio is often used as a proxy for placental efficiency^48^ and the decrease in this ratio may reflect aberrant placental function following in utero ZIKV infection, which is consistent with both our functional imaging data (Fig. 4) and the placenta pathology described below.

### Uteroplacental pathology

All five ZIKV-infected cases showed at least microscopic placental infarctions (Fig. 5a). Larger gross infarctions were visible in the cases infected earlier in gestation (Supplementary Fig. 7). Additionally, there was a subtle pattern of villous stromal calcifications compared with the gestational age-matched uninfected controls (Fig. 5b), which we concluded is most likely a feature of stromal fibroblast cell necrosis (Fig. 5c). Villous maturation was similar between cases and controls. Chorionic villi were negative for chronic villitis (feature sometimes seen in viral infections like CMV, or herpes), but ZIKV-infected cases did have diagnostic chronic decidualitis with plasma cells, which are required for the diagnosis. Two of the five cases had a pronounced leukocytoclastic vasculitis with a mixture of lymphocytes, plasma cells, and a few eosinophils infiltrating the spiral artery muscular wall (Fig. 5c, d). This type of vasculitis is usually related to a hypersensitivity reaction, but viral infections are also a well-recognized etiology^49^. In the three of the ZIKV cases infected at days 31–51, some of the spiral arteries also had less active inflammation, but clearly showed evidence of more remote lymphocytic vasculitis with medial damage and abnormal fibrin deposition (not part of the normal physiologic changes that characterize pregnancy-induced vascular remodeling (Fig. 5e, f)^50^. Spiral artery vasculitis was absent in the two ZIKV cases infected at 115dGA (these two cases also had fewer plasma cells in the decidua, fewer placental villous calcifications, and only microscopic placental infarctions), which likely reflects the shorter time between infection and tissue collection.

### MRI of the fetal brain

**Fig. 6:**
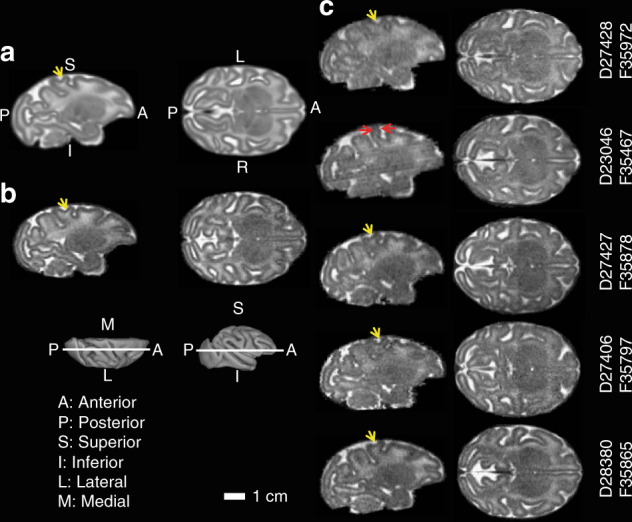
Magnetic resonance imaging of fetal brains. A T2W brain template generated from 16 control fetuses at gestation age 135dGA ± 2d (**a**), a typical control at 135dGA (**b**), and the 5 ZIKV-exposed fetal brains (**c**) are shown in sagittal (left) and axial (right) views at the indicated slice locations (insets). Anterior/posterior, superior/inferior, lateral/medial, and left/right are marked on the 3D brain surfaces (insets) and template images (**a**). Despite the absence of microcephaly, a thinner somatosensory gyrus (**c**, red arrows), and a missing secondary sulcus (**a**–**c**, yellow arrows), was identified for animal F35467, but not other ZIKV-infected animals

**Fig. 7:**
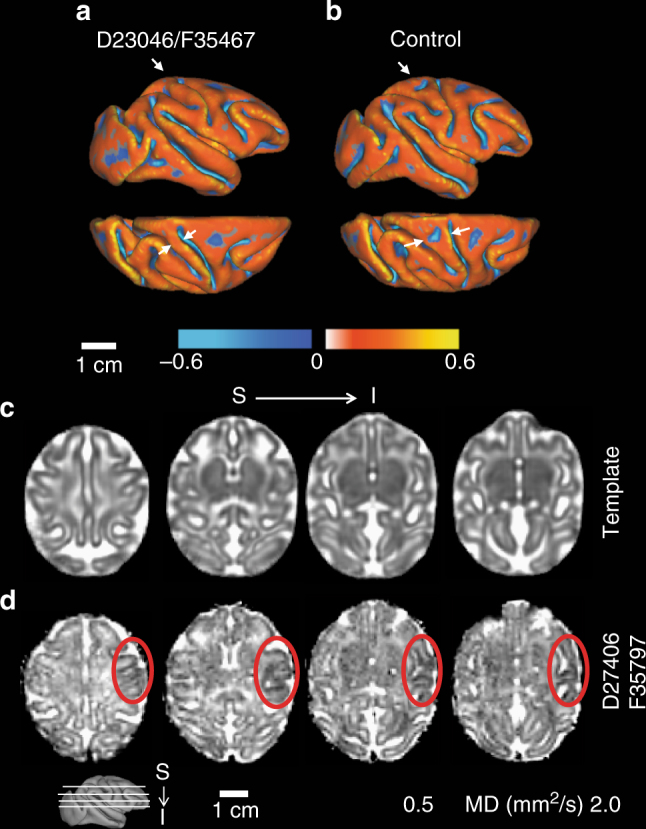
Cortical surface model and apparent diffusion coefficient (ADC) map of fetal brains. For animal F35467, a 3D cortical surface model was generated and curvature of the cortical surface was rendered on the surface (**a**, top, lateral view; bottom, dorsal view). Compared to an age-matched control (**b**), a significantly thinner postcentral gyrus, especially at the dorsal portion, can be detected (**a**, arrows). A missing secondary sulcus (**b**, asterisks) was also identified on the postcentral gyrus of animal F35467. **c** An ADC map generated from 8 control fetuses at gestation age 135dGA ± 2d and **d** one ADC map from ZIKV animal F35797 are shown at 4 axial slices at indicated levels (inset). Lower ADC is observable on the right hemisphere at the regions of motor, somatosensory, as well as auditory cortices compared to the left hemisphere (red dashed circles)

In spite of ZIKV-induced pathology and disruption of placental function, only minor effects on fetal brain development were observed by fetal MRI at 135dGA. To facilitate examining potential fetal brain abnormalities following ZIKV infection, T2W volumes of each ZIKV-infected fetal brain were linearly registered to a template generated from 16 age-matched experimentally-naive controls (manuscript in preparation). Sagittal (Fig. 6a) and axial views (Fig. 6a) at indicated levels (Fig. 6, insets) are shown for the template and a 135dGA experimentally naive fetus of an uninfected dam. Fetal brain T2W MRI are shown at corresponding locations for each ZIKV-infected animal in Fig. 6c. In contrast to human fetuses exposed in the first trimester^51^ and a pigtail macaque infected at 119dGA of a 162dGA term^27^, no obvious signal intensity anomalies within the cerebral cortical gray matter or fetal white matter of the T2W images were observed in the infected animal (Fig. 6a, b). Subtle abnormalities were observed in two of the fetal brains. A thinner postcentral gyrus, and a missing secondary sulcus was identified in animal F35467 (Fig. 6a, red and yellow arrows, note the “Y”-shaped post central gyrus in the template and other 4 ZIKV-infected fetal brains). To better characterize the abnormal gyral folding pattern detected in this animal, a 3D cortical surface model was generated and curvature of the cortical surface was rendered on the surface (Fig. 7a, top, lateral view; bottom, dorsal view), and an age-matched control (Fig. 7b). The surface area of the post-central gyrus, quantified as the percent of isocortex, for 10 age-matched controls fetuses studied previously^52^. In these animals, the post-central gyrus occupies between 4.4 and 5.7% of the isocortex (mean ± SD of 5.04 ± 0.45). The post-central gyrus for animal F35467 occupied 4.3% of the isocortex, which is smaller than all 10 control animals previously studied, and 1.6 SD smaller than the average value for controls. In animal F35797, reduced apparent diffusion coefficient (ADC) was observed in the right motor, somatosensory, and auditory cortices compared to the left hemisphere (Fig. 7d, red circles). The cortical regions marked by lower ADC were continuous throughout multiple levels (Fig. 7d). An ADC map template generated from 8 control fetuses at matched gestational age show homogeneous cortical ADC between hemispheres, and throughout the entire cerebral cortex (Fig. 7c). At necropsy, brain tissue from the low ADC regions (Fig. 7d, red circles) was dissected and determined to be positive for ZIKV RNA. Interestingly, F35797 was the fetus of dam D27406, the only ZIKV-infected animal with microvascular flux rate and placental oxygen permeability that was comparable to uninfected controls.

### Fetal pathology

**Fig. 8:**
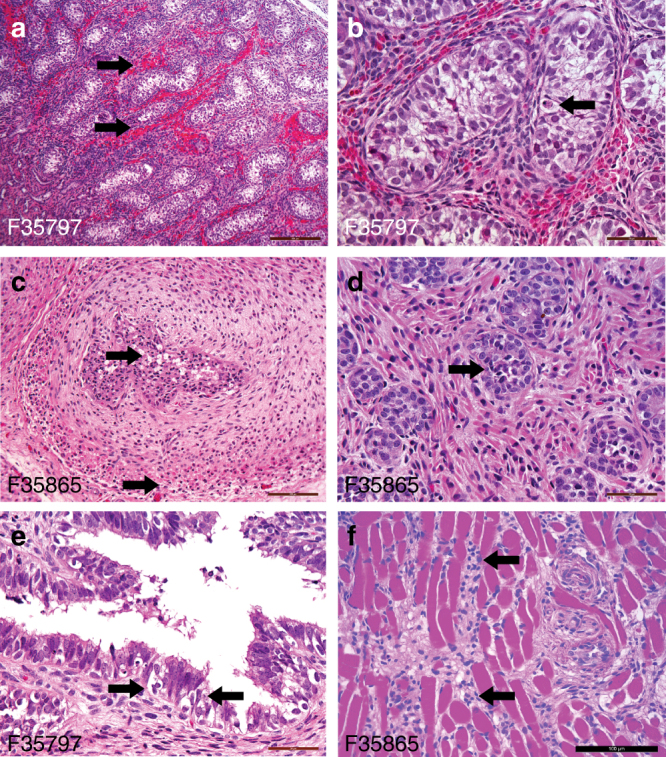
Histological images of fetal genitourinary tract tissues. At delivery, fetal tissues from testis (**a**, **b**), seminal vesicle (**c**), prostate (**d**), seminal vesicle (**e**), and pelvic floor (**f**) were paraffin-embedded, sectioned and stained with hematoxylin and eosin. Shown are representative images. Arrows indicate the presence of the following: **a** testicular hemorrhage; **b** testicular apoptotic cell; **c** seminal vesicle degeneration and apoptotic bodies in both the epithelial and smooth muscle layers; **d** vacuolar degeneration and apoptotic cells in the prostate; **e** seminal vesicle vacuolar degeneration and apoptotic bodies; and **f** lymphocytic infiltrates in the skeletal muscle of the pelvic floor adjacent to the bladder. Scale bar is 200 µm (**a**, **c**), 100 µm (**f**), and 50 µm (**b**, **d**, and **e**)

Histological assessment of the genitourinary tract revealed the presence of tissue and cellular damage in the female and male fetuses (Fig. 8). Neutrophilic inflammation (cystitis) of the bladder wall was identified in one animal (F35972, 31 dGA infection) and lymphocytic inflammation of the pelvic floor adjacent to the bladder was identified in one animal (F35865, 114 dGA infection). Interstitial hemorrhage was seen in sections of the testis from three animals (F35878, F35865, and F35797), indicating vascular damage following ZIKV infection. Individual apoptotic cells and cells undergoing vacuolar degeneration were present in testis, seminal vesicle, vas deferens, and prostate (Fig. 8). Damage in the seminal vesicle was generally focal and involved both the epithelial cells and the underlying smooth muscle cells. Similarly, abnormal focal interstitial hemorrhage was present in the cervix of F35467 (51 dGA infection) as well as smooth muscle cell degeneration. While we did not detect ZIKV RNA in all of the affected tissues, this could be due to the focal nature of infection, assay sensitivity or viral clearance by the time of necropsy. The collective observations of viral detection in the urine from all fetuses and some of the female and male fetal reproductive tracts combined with cellular and vascular damage to these tissues suggest that fetal genitourinary tracts are targeted by ZIKV and viral mediated pathogenesis.

## Discussion

We describe a translational nonhuman primate pregnancy model of ZIKV infection using a clinically relevant strain of ZIKV^53^. We demonstrate productive infection of five pregnant rhesus macaques at three different time points across gestation and draw comparisons to gestational-age-matched uninfected controls. We observed long-term persistence of virus in both maternal and fetal tissues, robust maternal and fetal innate and adaptive immune responses, and a systemic fetal inflammatory response to congenital infection with ZIKV. In addition, we report aberrant in vivo placental function secondary to ZIKV infection related to uteroplacental pathology. Notably, we did not observe fetal growth restriction or microcephaly in these animals. Importantly, initial reports from the WHO and CDC highlighted microcephaly as the major concern with vertical transmission of ZIKV infection in pregnancy. However, recent studies refer to a more inclusive clinical syndrome with microcephaly representing only one severe manifestation despite a range of adverse obstetric outcomes associated with ZIKV infection. In agreement with these studies, we detected neurological development abnormalities in two out of five fetuses in our ZIKV-infected cohort. Indeed, human epidemiological data suggest a spectrum of responses to ZIKV exposure during pregnancy^3,4^, and our data in pregnant rhesus macaques support and strengthen this finding with a range of fetal responses demonstrated following maternal ZIKV infection.

Previous studies by Nguyen and Dudley identified prolonged viremia following ZIKV infection in pregnant rhesus macaques relative to non-pregnant ZIKV-infected animals^21,28^. We did not observe this prolonged viremia in our study, which could be due to the lower sensitivity of our detection assays or the reduced frequency of blood sampling. While we cannot determine the precise timing of placental and fetal infection after subcutaneous maternal infection with ZIKV, detection of virus in the placenta and fetus in the animals infected at 115dGA and delivered 20dpi indicate that transplacental fetal infection can occur rapidly. We hypothesize that this process occurs during the early peak phase of maternal viremia. Viral RNA was detected in maternal serum as early as 3dpi and persists in maternal tissues and urine for extended periods, up to 85 dpi in this study, which is consistent with previous findings in non-pregnant rhesus macaques^22,26^. The persistence in maternal tissues could provide a reservoir facilitating vertical transmission of ZIKV. Furthermore, viral RNA in the maternal genitourinary tract, particularly the uterus, may be a concern when contemplating invasive diagnostic testing with amniocentesis and merits further investigation. Similarly, viral RNA was detected in the fetus and placenta at 85 dpi in spite of the immune response mounted by both the dam and fetus. The wide distribution of viral RNA in fetal tissues, similar to the maternal distribution, provides a foundation to interpret the varied phenotypes reported in the CZS, and provide a basis for understanding the broad range of developmental consequences that may result from CZS. While detection of ZIKV RNA in the urine from all fetuses, combined with the pathologic changes in the fetal genitourinary and reproductive tracts, suggests that fetal urinary and reproductive tracts are targeted by ZIKV and virus-mediated pathogenesis, the long-term pathologic consequences are potentially significant and warrant further investigation.

As other groups have recently reported with a different strain of ZIKV^28^, fetal infection occurred at all gestational time points, which is consistent with human reports^5^ of infection occurring both early and late in gestation. Uncertainty exists on the susceptibility of human trophoblasts to infection with ZIKV in vitro, with some reports suggesting greater placental tropism in first vs. third trimester placenta trophoblasts^30,35,54^. Our primary trophoblast cultures from ZIKV-infected dams demonstrate ZIKV E protein expression from animals infected at 31, 51 and 114 dGA, suggesting all are susceptible to infection. Furthermore, viral RNA was detected only in the primary trophoblast culture from 114 dGA infection, which also contained the most ZIKV E-expressing cells, possibly reflecting the short time elapsed between maternal infection and tissue collection (21 days). Human studies have suggested that vertical transmission may take 5 weeks; here we detected vertical transmission in two macaques infected at the end of the 2nd trimester within 20 days.

We comprehensively evaluated the interplay between the maternal-fetal and placental inflammatory milieu in response to ZIKV infection during pregnancy. Our data suggests that ZIKV exposure in utero produces a robust proinflammatory fetal immune response characterized by alterations in classical vs. non-classical monocyte phenotype and presence of activated CD4 T-cells as well as elevated cytokines (IL-12, IL-2 TNFα and IFNγ) and the chemokine IP-10 (CXCL-10). IP-10 is inducible by proinflammatory mediators such as interferon-γ (IFNγ), TNFα, and several viruses^55–58^. This chemokine is involved in the recruitment and potentiation of Th1 type inflammatory responses. The fetus is a semi-allograft, and active maternal tolerance mechanisms (Th2 and Treg recruitment) are critical to prevent fetal rejection. Inflammation of the placenta and decidua due to viral infections not only alters fetal organ development but also alters the fetal immune system, and can result in aberrant postnatal immune responses to infections^8,59^. The implication is that in utero ZIKV infection may cause vulnerability in the neonatal innate and adaptive immune responses and thus increase the susceptibility to subsequent sepsis, additional infections and autoimmune diseases.

**Fig. 9:**
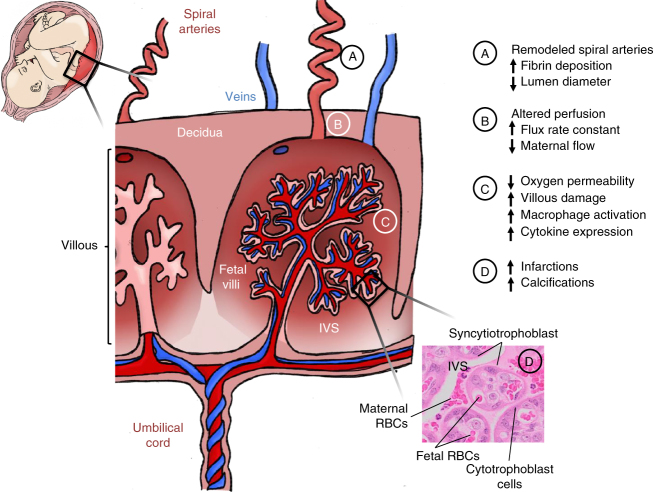
Schematic summary of placental damage following maternal ZIKV-infection. Cross section diagram of placental vasculature demonstrating maternal spiral arteries that perfuse the intervillous space. Notations A–D summarize the placental tissue damage, alterations in perfusion, and immune response to ZIKV infection during pregnancy

Functional imaging of the placenta in vivo by CEUS and MRI provide compelling evidence that ZIKV can alter placental function (summarized by the placental schematic in Fig. 9). First, the CEUS data in 4 of 5 ZIKV animals demonstrate an increased flux rate through the spiral arteries into the intervillous space. The microvascular flux rate data generated from our CEUS analysis represents the in-flow velocity of the contrast agent as it enters the intervillous space. This velocity is a component of flow but does not equate directly with blood flow, which requires a measure of volume; achieved in our studies by using DCE-MRI immediately following CEUS. Nonetheless, together with spiral artery histopathology, this increased rate of in-flow velocity could result in shear stress injury to the placental villi, which may explain the observed decrease in permeability-surface area product by functional MRI. Specifically, functional MRI demonstrated a higher level of oxygenated maternal blood within the placenta and decreased oxygen permeability-surface area product with ZIKV infection in the same 4 of 5 animals. The increased level of oxygenated maternal blood within the placenta could reflect decreased oxygen permeability across the villous trophoblast and/or decreased fetal metabolic demand. The decreased oxygen permeability-surface area product with ZIKV infection suggests that disruption in oxygen permeability across the villous trophoblast contributes to this higher-than-expected oxygenation of maternal blood within the placenta. The increased echogenic appearance observed with ultrasound that was also confirmed by high-resolution post-contrast MRI revealed placental lesions that were subsequently shown to be gross and microscopic infarctions and villous stromal calcifications. We suspect placental damage leads to decreased oxygen permeability, which may provide a mechanism for the observed association between ZIKV infection and adverse obstetric outcomes, including stillbirth, in some cases of CZS. The placenta pathology observed in our study recapitulates many features of pathology reported in post-delivery ZIKV-infected human placentas with villous calcification, stromal proliferation, and intravillous and perivillous fibrin deposition^60,61^; microscopic structural changes which could impact oxygen transport across the villi.

We did not detect IUGR in this model, the macaques were delivered at 135dGA, prior to the significant growth phase in the third trimester; as a result, we cannot exclude the possibility that fetal growth abnormalities may have been observed if the pregnancies had been taken to term. In one animal infected at 115 dGA, placental function both by CEUS and MRI was similar to control animals despite it demonstrating fetal infection similar to the other ZIKV-infected animals. This finding suggests that, while placental dysfunction could contribute or exacerbate fetal developmental disruption associated with infection, fetal infection can occur in spite of normal placental function in vivo. The placental infection to ZIKV is focal, which was determined only by comprehensive biopsy of all placental perfusion domains. Interestingly, the focal areas where virus was detected corresponded to the regions with decreased perfusion and gross infarctions in the one animal where three-dimensional isosurfaces were rendered.

Fetal ultrasound and biometry did not demonstrate evidence of microcephaly, which was confirmed with fetal MRI, and at delivery. Further, T2-weighted image hyperintensities in germinal zones or developing white matter, reported in other studies of ZIKV-infected nonhuman primates^27,28^, were not observed for any of the animals examined here. Although it is not possible to conclude that placental pathology is completely separable from brain injury because subtle fetal brain abnormalities were observed by in utero MRI in 2 of 5 infected animals, the overall absence of severe brain pathology, in the presence of perturbed placental function resulting from ZIKV infection in this model, is notable. In one animal (F35797), a region in the cerebral cortex with conspicuously low ADC was observed. Viral RNA was recovered from this area. In a second animal (F35467), an anomalous shape of the post-central gyrus and the absence of a minor sulcus immediately caudal to the dorsal central sulcus were observed. Although these cortical surface characteristics resulted in the formation of an extremely small post-central gyrus (smaller than 10 age-matched fetuses examined in a recent study^52^), this cortical shape anomaly is sufficiently subtle that the possibility it is a rare cortical folding variant cannot be excluded. In the 2 animals where CNS abnormalities were identified, the effects of infection appeared to be focal, as in the placenta. However, additional regions of infected brain cannot be excluded for all fetuses. The animal with the virally-infected cortical region of reduced water ADC was infected relatively late in pregnancy (115dGA), and placental function was normal, as assessed with in vivo imaging. This suggests that placental injury, as detected by our methods, is not a prerequisite to vertical transmission. The varying degrees of neurodevelopmental disruption reported in CZS thus may reflect damage resulting from inflammation and placental dysfunction when fetal metabolic requirements exceed the placental reserve capacity in addition to any direct tissue injury from ZIKV. Clarification of the relative roles of these two factors in CNS injury will require further study.

In summary, this work in a nonhuman primate model that shares developmental ontogeny with humans demonstrates that ZIKV infection during pregnancy results in a spectrum of feto-placental outcomes. Our findings not only highlight the need for further understanding of the causal mechanisms of ZIKV infection, but demonstrate the importance of redefining the clinical criteria and assessment of pregnant mothers suspected for infection. Improved antenatal monitoring may allow for modification of clinical management plans and reduce the adverse impact of maternal ZIKV infection on fetal and neonatal developmental consequences.

## Methods

### Ethics statement regarding nonhuman primate research

Zika virus infection of five pregnant rhesus macaques was performed in compliance with local and national animal welfare bodies and in strict accordance with Institutional Animal Care and Use Committee (IACUC) protocols. Rhesus macaque studies were performed in a bio-containment facility at the Oregon National Primate Research Center (ONPRC), which is accredited by the Assessment and Accreditation of Laboratory Animal Care (AAALAC) International. The dams and fetuses were humanely killed using a method that is consistent with the recommendation of the American Veterinary Medical Association.

### Zika virus PRVABC59

Zika virus isolate PRVABC59^22,53^ was generously provided by the Centers for Disease Control (CDC). PRVABC59 was passed twice on C6/36 cells (ATCC CRL-1660), and a working stock was concentrated by ultracentrifugation through a 20% sorbitol cushion and titered in Vero cells (ATCC CRL-1586). The working stock was sequenced and described previously^22^. All cells were cultured in Dulbecco’s modified eagle medium (DMEM) containing penicillin-streptomycin-glutamine and 5–10% fetal calf serum (FCS).

### Study design

Gestational age was determined by monitoring daily blood samples for the mid-cycle peak in estradiol, which immediately precedes the surge of luteinizing hormone responsible for ovulation. Females are paired with males several days prior to the estradiol surge and are then separated the day after peak levels are observed. Day 1 of gestation is considered 48 h post-estradiol surge. This timed-mating scheme allows for gestational age to be determined within 24–48 h of conception. At 31, 51, 114, and 115 days of gestation (dGA; term = 168 days) timed-pregnant rhesus macaques (*Macaca mulatta*) were infected subcutaneously with a total of 1 × 10^5^ focus-forming units (ffu) of ZIKV PRABC59 diluted in 1 ml of PBS that was distributed over both hands, wrists and upper arms as 10–100 µl injections (to mimic the typical pattern of mosquito bites)^22^. The dams were evaluated daily for clinical signs of disease. Body temperature along with peripheral blood and urine samples were obtained, and fetal/placental ultrasound analyses were performed at indicated times post infection (Fig. 1). Blood was collected on 0, 3, 7, 14, 21, 28, 35, 43, 49, 57, 63, 71, 78, 85, 91, 98, and 105 dpi with the following exceptions: (1) Animals D28380 and D27406 was taken to necropsy at 21 dpi; (2) Animals D23046 and D27427 were taken to necropsy at 85 dpi; and (3) day 3 was not collected for D23046. Collection of blood samples and ultrasound analysis were performed under ketamine sedation (10 mg/kg, IM). Peripheral blood mononuclear cells (PBMCs) and plasma were isolated by centrifugation over lymphocyte separation medium and then analyzed for lymphocyte phenotype and frequency by flow cytometry. Plasma was assessed for viral load by qRT-PCR and the levels of cytokines by a multiplex-bead based assay. Urine and saliva were assessed for viral RNA by qRT-PCR and for infectious virus by co-culture and focus-forming assays using Vero cells. An in utero MRI scan of the fetal brain and placenta was performed just prior to delivery. Necropsy was performed on the all of the dams and fetuses at 135dGA. Samples of maternal and fetal tissues (joints, muscles, organs, brain, spinal cord, eyes, glands, lymph nodes and bone marrow) and biological fluids (cerebral spinal fluid, blood, amniotic fluid, and urine) were collected and stored in RNAlater, Trizol (RNA isolation), medium (virus isolation) as well as formalin fixed and embedded in paraffin.

### ZIKV detection by one-step qRT-PCR analysis

RNA from maternal, fetal and placental tissue samples, blood, urine, CSF, and amniotic fluid was isolated using Trizol (Invitrogen) according to manufacturer’s protocol. ZIKV RNA levels were measured by a one-step quantitative real-time reverse transcription polymerase chain reaction assay (qRT-PCR) using TaqMan® One-Step RT-PCR Master Mix (Applied Biosystems). Primers and probes were as previously described^62^ with a one base change in the probe to match the PRVABC59 sequence (Genbank accession #: KU501215.1). Forward primer: 5′- CCGCTGCCCAACACAAG-3′ (ZIKV PRVABC59 genome sequence nucleotides 1192–1208); reverse: 5′- CCACTAACGTTCTTTTGCAGACAT-3′ (complement of nucleotides 1245–1268); and TaqMan probe: 5′ Fam-5′-AGCCTACCTTGACAAGCAATCAGACACTCAA-3′ -MGB (nucleotides 1213–1243). Forward and reverse primers were used at 250 nM in the reaction, and the probe at 200 nM. The limit of detection for our One-step RT-PCR quantification assay was approximately 100 genomes making the limit of detection in bodily fluids 1e4 for 100 µl of plasma or urine and 1/10th of the RNA recovered used per reaction. The limit of detection in tissues was ~400 genomes per µg total RNA using 250 ng total RNA per reaction.

### Isolation of placental immune cells and trophoblasts

At the time of necropsy, ~0.5–1 g of decidua and villous tissue were collected and stored in 5 mL of HBSS supplemented with 2% FBS and 10 mM Hepes (HBSS + ), for flow cytometry analysis. Tissues were dissected into a fine slurry using forceps and scissors prior to being digested with collagenase, 50 mg collagenase in 35 mL HBSS + , for 30 min at 37 °C, with rocking. Digested tissues were washed with HBSS + and run through a 70 μM filter. The cells present in the flow through were pelleted by centrifugation at 500 × *g*. Red blood cells were lysed in the cell pellet with BD Pharm Lyse lysing buffer (BD Biosciences). After a final wash in HBSS + , cells were resuspended in 5 mL HBSS + and counted using a hemocytometer. Cells were analyzed by flow cytometry as described below.

To evaluate placental infection by ZIKV, villous trophoblasts were isolated from the placentas of three animals (D27428, D27427 and D28380) essentially according to the method of Kliman et al.^63^. Briefly, villous tissue (30^–^35 g) that had been dissected free of membranes was washed, minced, filtered through sterile gauze and digested with three rounds of incubation in a trypsin-DNase solution (0.25% trypsin [Sigma-Aldrich, St. Louis MO] in PBS with 0.5 mg/ml DNase 1 [Sigma; 2000 Kunitz units/mg]). Digested tissue was collected, layered over newborn calf serum (NCS; Sigma) at a 5:1 v/v ratio and centrifuged at 1000 × *g* for 5 min. Cell pellets were resuspended in DMEM and the mononuclear cytotrophoblast population was isolated by centrifugation on a preformed Percoll gradient (10–70%) with collection of cells banding between 35–55%. Recovered cells were washed and plated on 35 mm Corning Primaria tissue culture dishes (Thermo-Fisher Scientific; Waltham, MA) in 1:1 DMEM/Hams F12 (Thermo-Fisher Scientific) supplemented with 10% FBS, 2 mM L-glutamine and antibiotics (Penicillin-streptomycin-neomycin). Cells were cultured for 3 days post plating (pp) with medium exchange every 24 h. Supernatants were harvested at days 1, 2, and 3 pp and evaluated for the presence of infectious ZIKV by titration on Vero cells. Monolayers were used immediately after each supernatant collection on days 1–3pp for immunofluorescence detection of ZIKV antigen. Briefly, cells were washed, fixed and permeabilized in 4% pFormaldehye followed by 0.2% Triton X-100, blocked with 20% normal goat serum and sequentially incubated with an anti-Flavivirus group antigen mAb (Clone D1–4G2) and a goat-anti-Mouse IgG Alexa-Fluor 488 (Thermo-Fisher) secondary antibody.

### Phenotypic analysis of peripheral blood and tissue mononuclear cells

Flow cytometry was used to quantify the immune cell phenotype and level of cellular proliferation and activation for maternal PBMCs isolated at the time points defined above as well as the immune cells obtained from placental tissues. The panel of antibodies used for the analysis of innate immune cells consisted of HLA-DR (G46–6 10 µg/mL Beckman Coulter), CD14 (HCD14 10 µg/mL Biolegend), CD11c (3.9 10 µg/mL Biolegend), CD123 (SSDCLY107D2 10 µg/mL Beckman Coulter), CD20 (B9E9 10 µg/mL Beckman Coulter), CD3 (SP34-2 2 µg/mL Biolegend), CD8 (SK-1 10 µg/mL BD Bioscience), CD16 (3G8 10 µg/mL ThermoFisher), and CD169 (7–329 2 µg/mL Biolegend). To differentiate between monocyte/macrophages, dendritic cells (DC), and natural killer (NK) cells the following gating strategy was utilized: monocyte/macrophages (CD3^−^CD20^−^CD14^+^HLA-DR^+^), classical monocytes (CD3^-^CD20^-^ HLA-DR^+^ CD14^+^CD16^−^), intermediate monocytes (CD3^−^CD20^−^ HLA-DR^+^ CD14^+^CD16^+^), non-classical monocytes CD3^−^CD20^−^ HLA-DR^+^ CD14^−^CD16^+^), DC (CD3^−^CD20^−^CD14^−^HLA-DR^+^), myeloid DCs (CD3^−^CD20^−^CD14^−^HLA-DR^+^ CD123^−^), CD11c^+^ plasmacytoid DCs (CD3^−^CD20^−^CD14^−^HLA-DR^+^CD123^+^CD11c^−^), other DCs (CD3^−^CD20^−^CD14^−^HLA-DR^+^CD123^−^CD11c^−^), and NK cells (CD3^−^CD20^−^CD8^+^CD16^+^). The percentage of activated cells (CD169^+^) within each subset was calculated as a representation of the cellular activation profile. T cells were analyzed with the following panel of antibodies directed against CD3 (SP34-2 2 µg/mL Biolegend), CD4 (L200 5 µg/mL Thermofisher), CD8β (SK-1 10 µg/mL BD Bioscience), CD95 (DX2 2 µg/mL BD Biosciences), CD28 (CD28.2 2 µg/ml Biolegend) and the intracellular proliferation marker Ki67 (20Raj1 2 µg/ml, Thermofisher). CD3^+^ T cells were first identified as CD4^+^ or CD8^+^; within the CD4^+^ and CD8^+^ T-cell subsets the naive (CD28^+^CD95^−^), central memory (CD28^+^CD95^+^), and effector memory (CD28^−^CD95^+^) subsets were analyzed. B cells were stained using the following antibodies: CD20 (B9E9 5 µg/ml Beckman Coulter), CD27 O323 5 µg/mL Biolegend), and IgD (Goat polyclonal 2 µg/mL Southern Biotech) to delineate naive (CD20^+^CD27^−^IgD^+^), memory (CD20^+^CD27^−^IgD^−^) and marginal-zone like B cells (CD20^+^CD27^+^IgD^+^) as well as Ki67 to identify recent proliferating cells. The percentage of proliferating (Ki67^+^) B and T cells within each subset was calculated. The gating strategies were performed as previously described^64^. Phenotyping was performed using an LSRII instrument (BD Bioscience) and the data were analyzed with FlowJo Software (TreeStar).

### ZIKV enzyme-linked immunosorbent assay (ELISA)

Anti-ZIKV antibodies were measured by ELISA in the maternal and fetal plasma as well as in the cord blood (venous and arterial) and amniotic fluid. High-binding polystyrene 96-well plates (Corning) were coated overnight with 100 µl of PBS containing a 1:100 dilution of purified ZIKV particle preparations. The plates were blocked with PBS containing 2% milk and 0.05% Tween (ELISA-Block) for 1 h at room temperature, washed with 0.05% Tween-PBS (ELISA-Wash), and incubated with two-fold dilutions of rhesus plasma in ELISA-Block starting at a dilution of 1:50. The plate was incubated at room temperature for 2 h. Plates were washed several times with ELISA-Wash and then incubated with secondary anti-monkey IgM/A/G (Rockland, Inc.) conjugated with horseradish peroxidase for 30 mins. Plates were washed with ELISA-Wash and bound secondary antibody was detected using the OPD substrate (Life Technologies) with an HCl stop. The plates were read within 10 min using a Synergy HTX Microplate Reader (BioTeck) at 490 nm. Dilution titers of ZIKV-binding antibodies were determined using a Log-to-Log transformation method and the results were graphed using GraphPad Prism v6 software.

### Anti-ZIKV neutralization assay

Plaque Reduction Neutralization Test (PRNT_50_) was used to measure the concentration of serum that neutralizes 50% of a fixed number of ZIKV-PRABC59. NHP serum from the dam and fetus, cord blood, and amniotic fluid was serially diluted 2-fold from starting dilutions of 1:10 and mixed with an equal volume of a fixed number of plaque forming units (50 PFU) of ZIKV for final serum dilutions ranging from 1:40 to 1:81,920. Sera and virus were incubated for 1 h at 37 °C. The mixtures were added to individual wells of 12-well plates seeded with Vero cells at 90% confluence for 1 h at 37 °C on a rocker and then overlaid with DMEM containing methylcellulose. Plates were incubated for 3 days and then fixed and counterstained with methyl blue. Plaques were counted and PRNT_50_ values were calculated utilizing the sigmoid dose-response curve fitting function of GraphPad Prism v6 software with upper and lower limits of 100 and 0, respectively.

### Cytokine assay

Luminex monkey Cytokine Magnetic 29-plex panel (Invitrogen) was used to quantify cytokine and chemokine expression in maternal blood and fetal (circulation and venous/arterial cord blood) plasma, amniotic fluid and CSF samples. The assay was performed according to the manufacturer’s instructions. Briefly, washed antibody-conjugated polystyrene magnetic beads were incubated with a 7-point standard curve or 25 µl of rhesus monkey plasma or CSF plus 25 µL of blocking buffer, and incubated 2 h. Beads were washed with wash buffer and labeled with the biotinylated detector antibody for 1 h. Beads were washed and then incubated with Streptavidin conjugated to R-Phycoerythrin for 30 min and washed. Cytokines were identified and quantified using a Luminex 200^TM^ Detection system (Luminex) and data was graphed using GraphPad Prism v6 software.

### Doppler ultrasonography

Standard Doppler ultrasound measurements for uteroplacental hemodynamics were performed by one ultrasonographer (AEF) as previously published for use with macaques^41,43,65–67^. Briefly, image-directed pulsed and color Doppler equipment (GE Voluson 730 Expert) with a 5-to9-MHz sector probe was used for ultrasonographic data collection. Blood velocity waveforms were obtained from the maternal uterine artery (UtA) and umbilical artery to calculate their pulsatility index (PI = [peak systolic velocity—end diastolic velocity]/time-averaged maximum velocity over the cardiac cycle) values. The mean velocity from the intra-abdominal umbilical vein (UV) was also obtained. The diameter of the uterine artery and intra-abdominal umbilical vein was obtained as previously described and utilized to calculate the uterine artery volume blood flow and umbilical vein volume blood flow^41,43,65^. Fetal head and abdominal circumference (HC, AC), bipartiel diameter (BPD) and femur length (FL) was assessed by ultrasonography to monitor potenial pertubationas in normal fetal growth and development (microcephaly or calcifications in the fetal brain and placenta).

### Contrast-enhanced ultrasound

At gestational day 135, following an overnight fast, animals were sedated by intramuscular injection with 10 mg/kg Ketamine. Animals were then intubated and maintained under anesthesia with 1–2% inhaled isoflurane gas for the duration of each imaging study. Contrast-enhanced ultrasound was performed using a multiphase amplitude-modulation and phase-inversion algorithm on a Sequoia system (Siemens Medical Systems, Mountain View, CA) as previously described in detail^47^. Lipid-shelled octofluoropropane microbubble contrast reagent (Definity®, Lantheus Medical Imaging, Billerica, MA) was prepared in 0.9% saline at a final concentration of 5% for intravenous infusion via a cephalic catheter. The acoustic beam was centered over individually identified maternal spiral artery sources and the microbubbles within the path of the beam were destroyed by a brief (2 s) increase in mechanical index. Microbubble re-entry in the spiral artery and the IVS was recorded at 1 frame /75 ms until the area of interest reached signal saturation (VImax). Digital video clips were recorded for 10 s durations to measure video intensity (VI) of the blood pool (VIpool). Three replicates of all recordings were obtained during each study. The digital imaging data were analyzed using a custom-designed CE-US analysis program as previously described^47^. In brief, regions of interest were drawn over the area of each placental cotyledon. The data were fit to the function *y* = *A*(1-e-*βt*), where *y* is the VI at the pulsing interval *t*, *A* is the VI plateau and *β* is the flux rate constant. A one-way ANOVA with Tukey’s post hoc multiple comparison test was used to compare microvascular flux rate data.

### Magnetic resonance imaging

MRI studies were performed on a nonhuman primate-dedicated 3T Siemens TIM-Trio scanner (Erlangen, Germany) using a circularly-polarized (CP) transmit, 15-channel receive radiofrequency (RF) “extremity” coil (QED, Cleveland, OH). ZIKV animals were transported under 1–2% inhaled isoflurane immediately from the ultrasound studies to the MRI facility. For fetal brain imaging, a half-Fourier acquisition single-shot turbo spin-echo (HASTE) sequence was used to acquire T_2_-weighted volumes (T2W) along the axial direction (relative to fetal brain). Four averages of T2W volumes were collected with TR/TE = 1200/102 ms at a resolution of 0.5 × 0.5 × 0.8 mm. A diffusion-weighted, 2D spin-echo-based EPI sequence was used to acquire one image volume with *b* = 0 (the “b0” volume) and 20 diffusion-weighted volumes with *b* = 500 s/mm^2^ along the fetal axial direction. Up to 6 averages of b0 and diffusion-weighted volumes were acquired with a resolution of 1.1 × 1.1 × 1.7 mm. Apparent diffusion coefficient (ADC) maps were calculated from b0 and diffusion-weighted volumes using the “DTIFIT” function within FSL software package (https://fsl.fmrib.ox.ac.uk/fsl/fslwiki). T2W volumes and ADC maps of ZIKV-infected animals were linearly registered to a T2W template and an ADC map template, respectively. The T2W template was generated from 16 control animals at gestational age G135 ± 2d, and the ADC map template was generated from 8 control animals at the same gestation age range (manuscript in preparation). For an animal shown abnormal gyral folding pattern and an age-matched control, 3D cortical surface models of the fetal brains were generated from manual segmentations of T2W volumes using CARET software (http://brainvis.wustl.edu/wiki/index.php/Caret). Cortical surface curvature (a metric for brain folding) were computed and rendered onto the 3D surfaces. Cortical gyri have positive curvatures (Fig. 7, warm colors) and sulci have negative curvatures (Fig. 7, cold colors). For placental MRI, following localization of the placenta and acquisition of T_2_-weighted HASTE anatomic images in the coronal and axial planes, axial 2D multislice multiecho spoiled gradient echo (SPGR) images spanning the entire uterus, were acquired as previously described in detail^36,37^. Subsequently, 3D SPGR images were acquired in the coronal plane, also covering the entire uterus, to allow estimation of *T*1 (longitudinal relaxation time) using the variable flip angle (VFA) method^37^. Immediately after acquisition of VFA data, 150 volumes of 3D SPGR images were acquired for DCE-MRI with intravenous injection of a standard dose of 0.1 mmol/kg of gadoteridol CR (Prohance, Bracco Diagnostics Inc, Princeton, NJ) at a rate of 30 mL/min using a syringe pump (Harvard Apparatus, Holliston, MA), followed by 3D post-contrast SPGR imaging. Anatomic and multiecho imaging was performed during expiratory breath-holding, achieved by temporarily suspending ventilation, while the DCE-MRI data were acquired during ventilation. Physiological monitoring of pulse rate, arterial blood oxygen saturation, and end-tidal CO_2_ partial pressure was performed throughout the imaging study, with no deviations from normal ranges observed in these parameters. Immediately following MRI, the animals were transported to the ONPRC Surgical Services Unit for Cesarean section. Subsequent to necropsy, the right hemisphere was immersion fixed in 4% paraformaldehyde and imaged in phosphate-buffered saline following previously described methods^37^.

### Histological analysis of placental tissue

Formalin-fixed paraffin-embedded tissue blocks representative of the placenta (8–11 separate 0.5 cm^3^ samples/placenta) and the placental bed emphasizing grossly visible spiral arteries (3–4 separate biopsies per case) provided hematoxylin and eosins stained (H&E) histologic sections for review by a gynecologic pathologist. Sections from five ZIKV cases and three gestational age-matched negative controls (135dGA) were reviewed while blinded to exposure group scoring for the presence or absence of placental infarctions, accelerated villous maturation, villous stromal calcifications, chronic villitis, acute chorioamnionitis, chronic decidualitis (highlighted by CD138, B-A38 cat#760-4248) immunostaining of plasma cells), and macrophage subtyping by CD68 (M1, KP-1 cat#790-2931) and CD163 (M2, MRQ-26 cat#760-4437) in villi and decidua. Antibodies were received pre-diluted (and ready for use and immunostains were performed on a Benchmark XT autostainer (Ventana Medical Systems, Tucson, AZ, USA). The number of plasma cells and macrophage subtypes were scored as number per high power field (40× objective) and reported as the average of the four most populated fields. Complete cross-sections of decidual spiral arteries were scored for normal luminal dilation and for the presence or absence of vasculitis.

### Fetal pathology

Formalin-fixed paraffin-embedded tissue blocks representative of the fetal tissues were stained with H&E followed by microscopic examination. Sections were reviewed by ONPRC veterinary pathologists.

### Data availability

All the relevant data are available from the authors.

## Electronic supplementary material

Supplementary Information
