# Supplementary material for: Zika virus infection in pregnant rhesus macaques causes placental dysfunction and immunopathology
Source: Nat Commun. 2018 Jan 17;9:263. doi: 10.1038/s41467-017-02499-9 (PMC5772047; doi:10.1038/s41467-017-02499-9)
Supplement: Supplementary file 1 — Supplementary Information [file 41467_2017_2499_MOESM1_ESM.pdf]

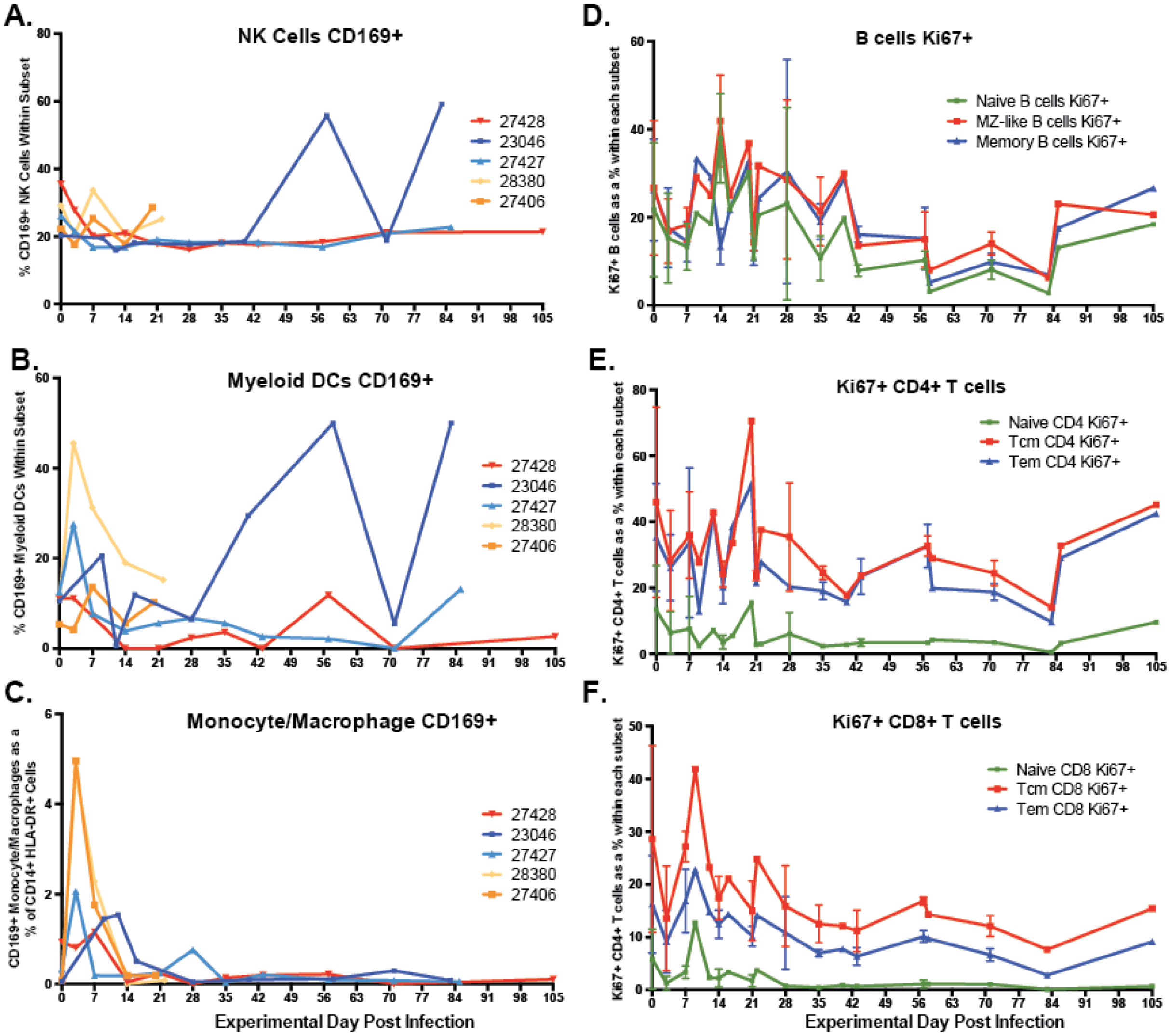

Supplementary Figure 1. Maternal Innate and Adaptive Immune Cell Proliferative Responses.
Innate Immune Cell Responses. Total maternal PBMCs from all time points were stained with fluorophore-conjugated antibodies directed against the cellular markers CD3, CD8, CD11c, CD14, CD16, CD169 and HLA-DR in order to assess changes in the activation of A) NK cells; B) myeloid dendritic cells; C) Monocyte/Macrophages. Multi-color flow cytometry was used to visualize the stained cells. The percentage of activated cells ($CD169^{+}$) was calculated using FlowJo.
Adaptive Immune Cell Responses. Total PBMCs were analyzed by flow cytometry for the presence of B and T cell proliferative responses following infection. D) B cells were stained with antibodies directed against CD3, CD20, CD27, IgD and HLA-DR as well as Ki67 in order to compare the proliferative responses in naïve, memory, and marginal zone-like (MZ-like) B cells. T cells were identified by staining with antibodies directed against the cellular markers CD3, CD4, CD8β, CD95, CD28, CD127 and for intracellular levels of Ki67 (proliferation marker) to assess changes the proliferation of E) $CD4^{+}$ naïve, central memory, and effector memory T cells; F) $CD8^{+}$ naïve, central memory, and effector memory T cells. The percentage of actively proliferating cells ($Ki67^{+}$) was calculated using FlowJo. Error bars represent the standard error of the mean.

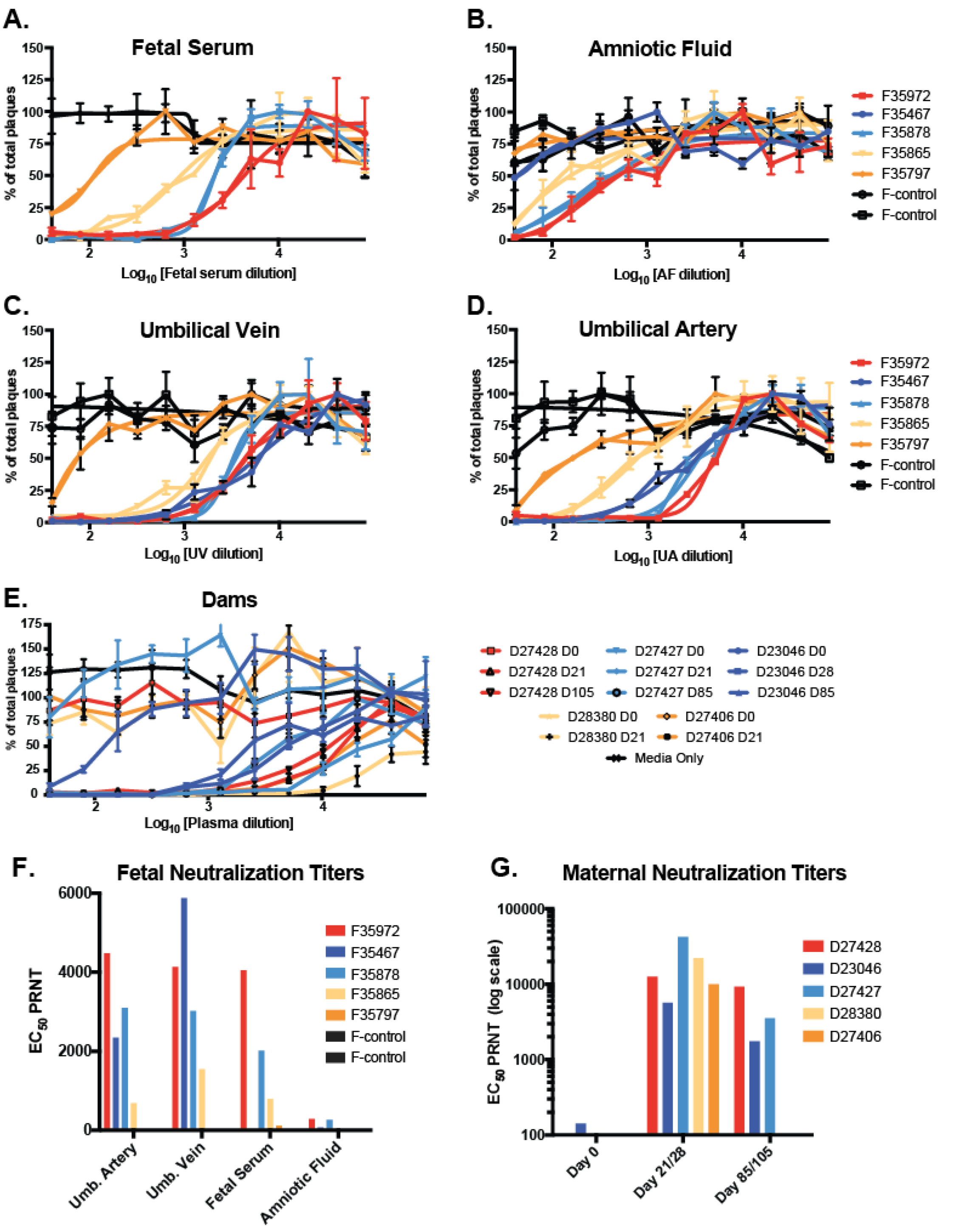

Supplementary Figure 2. Detection of Anti-ZIKV Antibody Responses in Maternal and Fetal Circulation. Viral plaque neutralization titer assays (PRNT50) were utilized to quantify anti-ZIKV antibodies present in fetal serum (A), amniotic fluid (B), plasma from umbilical vein (C) and artery (D) collected at delivery and in maternal circulation (E) at times indicated. Graphical representation of combined fetal (F) and maternal (G) PRNT data. Error bars represent standard error of the mean.

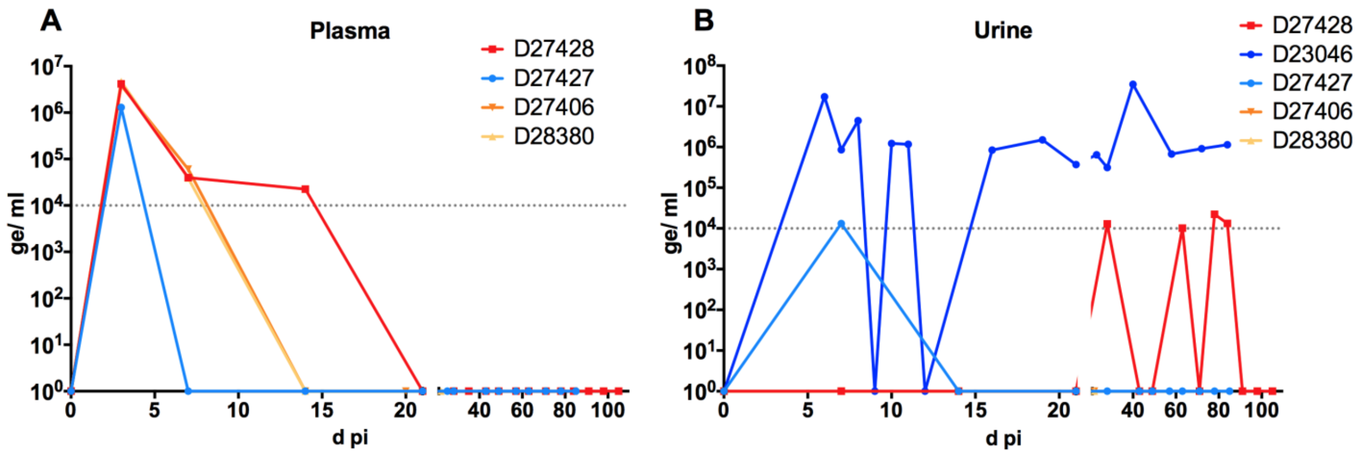

Supplementary Figure 3. Viral loads in maternal plasma and urine. One-step qRT-PCR was used to measure ZIKV RNA loads in the plasma (A) and urine (B) from the pregnant dam at indicated days post infection and represented as copies per milliliter of urine. A tenth of the total RNA extracted from 100µl urine was used in each reaction. Approximate limit of detection at 1e4 genomes/ml is based on a detection limit of ~100 genomes in each reaction.

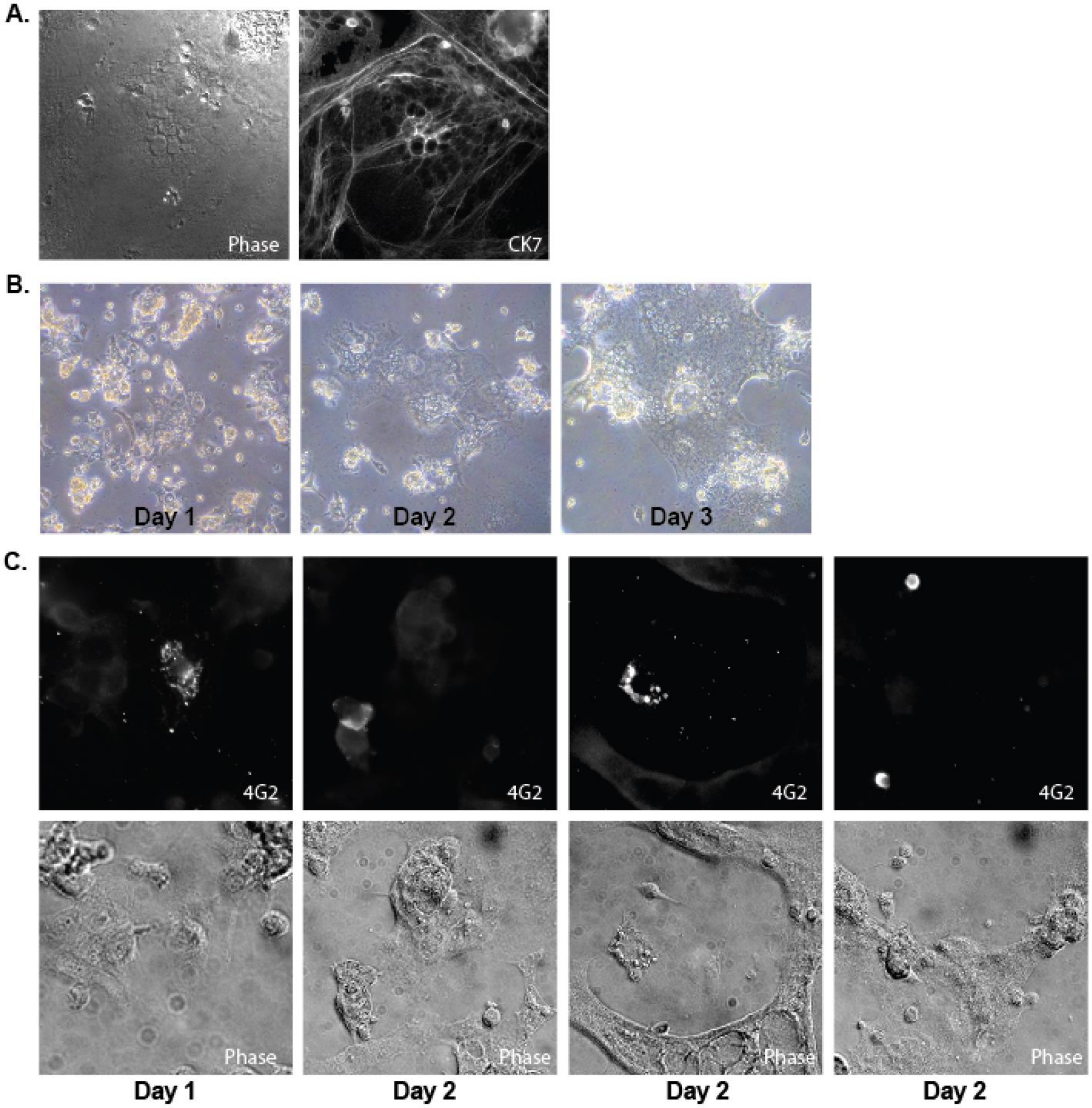

Supplementary Figure 4. Isolation and Analysis of *Ex Vivo* Placental Trophoblast Cultures. Trophoblasts isolated from placental villous tissue by enzymatic digestion and Percoll gradient fractionation were stained for cytokeratin 7 to confirm trophoblast enrichment (A), evaluated by phase microscopy on 1, 2 and 3 to illustrate the presence of mononuclear CTB with fusion to differentiated STB (B) on days 1 through 3 post plating (days post plating, dpp), and evaluated for the presence of cells containing ZIKV E antigen by immunofluorescent staining with the 4G2 mAb (C). Panel C shows representative dark and phase images of cultures derived from animal D28380. The far and center left panels depict infected trophoblasts at 1dpp (far left) and 2dpp respectively. The center right and far right panels depict infected cells with the morphology of HC and lymphocytes respectively.

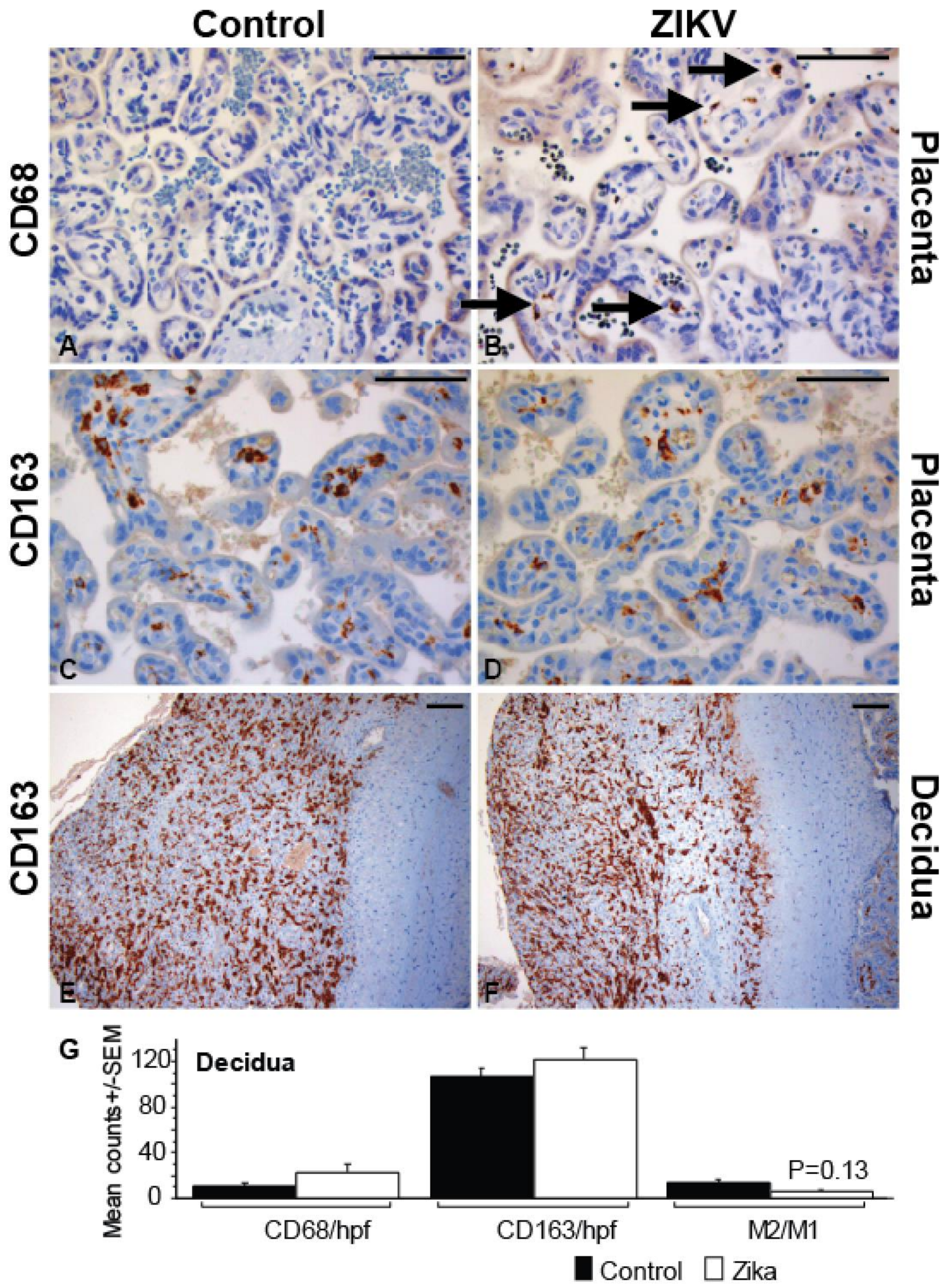

Supplementary Figure 5. Macrophage Polarization in ZIKV Infected Cases Compared with Controls. Pro-inflammatory CD68 positive M1 macrophages that promote antiviral Th1 CD4-mediated responses are conspicuously present in the chorionic villi and decidua of ZIKV cases, but not controls. CD163 positive M2 macrophages that contribute to tissue repair and promote inhibitory T helper responses (Th2) are abundant in the chorionic villi and decidua of both ZIKV cases and controls. Despite the relatively few cases tested, there is a trend towards more M1 polarization in the ZIKV cases. Scale bar is 100um.

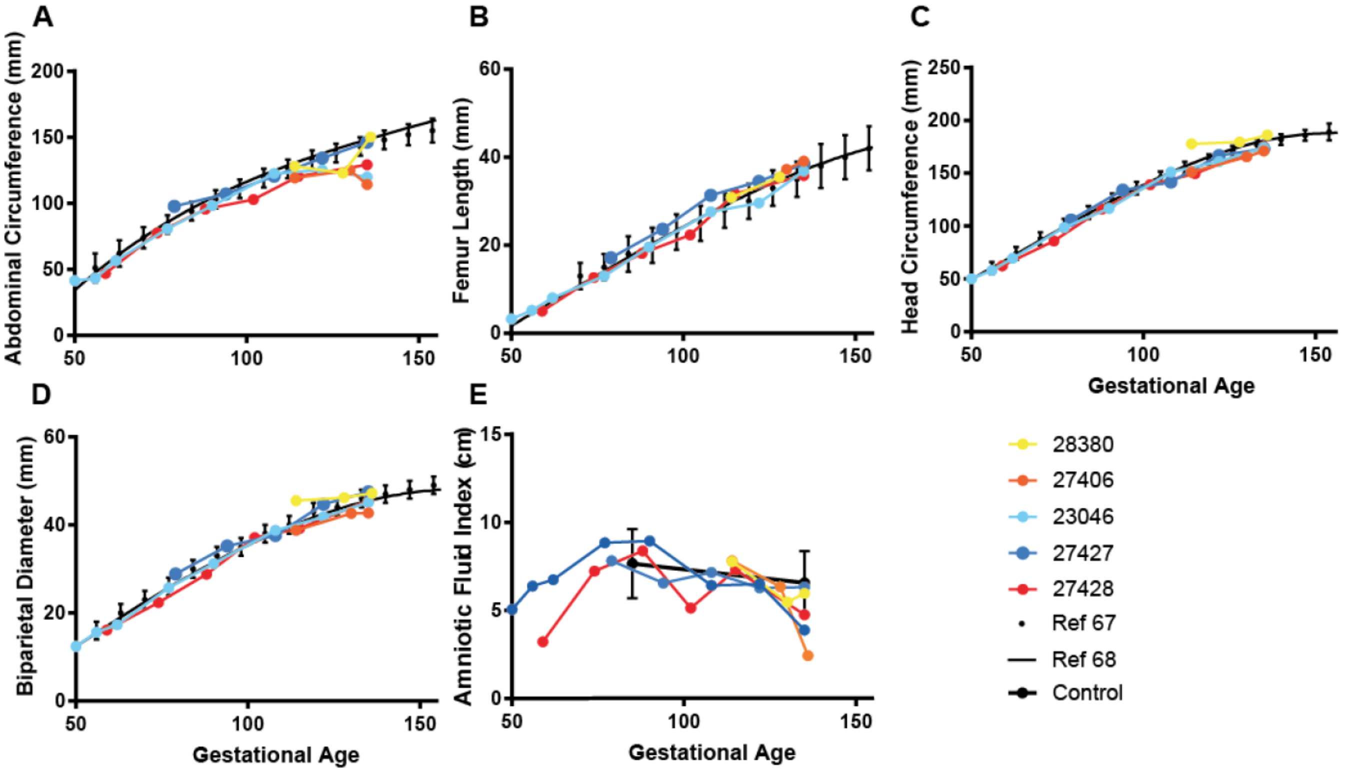

Supplementary Figure 6. Fetal Growth and Amniotic Fluid Measurements. Serial ultrasound measurements of fetal growth across gestation including, A) Abdominal circumference, B) Femur Length, C) Head circumference, and D) Biparietal Diameter in the five ZIKV-infected fetuses plotted against two reference data sets; one published macaque study [1] and a calculated prediction of rhesus growth [2]. E) Amniotic Fluid Index data obtained by standard measurement of four quadrants with control data (black closed circles) from non-infected animals (n=6) and individually plotted ZIKV-infected animals.

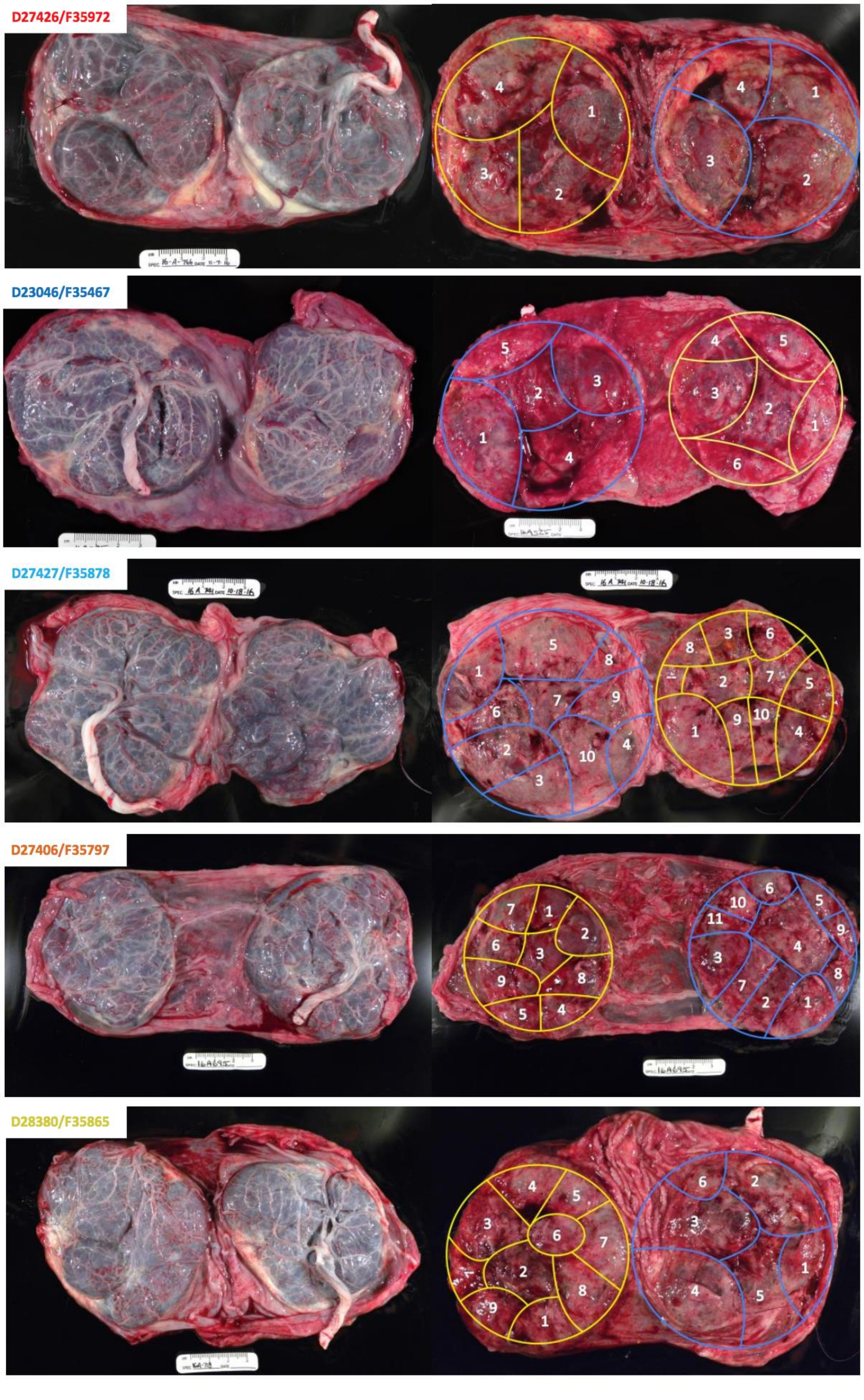

Supplementary Figure 7. Gross Placenta Pathology. Fetal facing (left panels) and maternal facing (right panels) photographs of the five ZIKV-infected placentas. Individually mapped cotyledons are annotated and numbered in the color overlays of the primary (blue) and secondary (yellow) placental lobes. Scale in centimeters.

	Animal ID	D27428	D23046	D27427	D27406	D28380
	infection (dGa)	31	51	51	115	114
lymphoid	Axilliary LN	nd	nd	nd	4.5	2.8
	Cervical LN	nd	nd	nd	nd	nd
	Inguinal LN	nd	nd	nd	3.2	3.7
	Mesenteric LN	nd	nd	nd	3.1	2.7
	Retroperitoneal LN	nd	nd	nd	nd	nd
	Salivary LN	nd	nd	nd		2.5
	Spleen	nd	nd	nd	3.8	3.4
	Thymus	nd	nd	nd	4.4	3.1
	Tonsils	nd	nd	nd	nd	nd
cardio-pulmonary	Heart	nd	nd	nd	nd	2.3
	Lung	nd	nd	nd	nd	nd
digestive	Gall bladder	nd		nd	nd	nd
	Liver	nd	nd	nd	nd	2.5
	Pancreas	nd		nd	2.7	nd
	Parotid salivary gland	nd	nd	nd	nd	nd
	Submandibular gland	nd	nd	nd	2.6	2.9
musculo-skeletal	Ankle	nd	3.3	nd	2.4	nd
	Bicep	nd	nd		nd	3.4
	Brachioradialis	nd		nd	nd	3.0
	Elbow	nd		nd	nd	nd
	Finger	nd		nd	4.0	2.7
	Hamstring	nd	3.1	nd	2.5	nd
	Knee	nd	3.0	3.4	nd	
	Quadriceps	nd	nd		nd	2.6
	Skin upper torso	nd	nd	2.9	nd	nd
	Soleus	nd	3.2	nd	nd	2.6
	Toe	nd	3.3	nd	2.8	nd
	Tricep	nd	3.2	nd	nd	nd
	Wrist	nd		nd	nd	3.7
genito-urinary	Bladder	nd	nd	nd	nd	nd
	Cervix	nd	nd	nd	nd	nd
	Kidney	nd	nd	nd	nd	nd
	Ovary	nd	3.1	nd	2.5	2.7
	Uterus	nd	nd	nd	nd	nd
	Vagina	nd	3.1	nd	nd	2.8
neuro-endicrine	Adrenal gland	nd	nd	nd	nd	nd
	Basal Ganglion	nd		nd		nd
	BR Cerebellum	nd	nd	nd	nd	nd
	BR Frontal lobe	nd	nd	nd	nd	nd
	BR Occipital lobe	nd		nd		3.0
	BR Parietal lobe	nd		nd		nd
	BR Stem	nd		nd		nd
	BR Temporal lobe	nd		nd		2.6
	Brachial plexus	nd	nd	nd	nd	nd
	Dorsal root ganglion	nd		nd	nd	nd
	Eye	nd	nd	nd	nd	nd
	Femoral nerve	nd		nd		nd
	Pituitary gland	nd	3.5	3.3		nd
	SC Cervical	nd	nd	nd		3.9
	SC Lumbar	nd	nd	nd	nd	2.5
	SC Thoracic	nd	3.4	nd	nd	nd
	Sciatic nerve	nd		nd	nd	nd
	Thyroid	nd	nd	nd		
	Trigeminal ganglion	nd		nd	2.4	2.7

Supplementary Table 1. Viral RNA loads in maternal tissues. Indicated tissues were analyzed by qRT-PCR for ZIKV RNA. Numbers (colored backgrounds) indicate $\log_{10}$ ZIKV genomes/ µg total RNA. BR: brain; LN: lymph node; nd: not detected; blank cells indicate tissue not present or not collected.

Fetus	Infection (dGA)	Urine	AF	CSF	UV	UA	Plasma
**F35972**	31	+	-	+	-	-	-
**F35467**	51	+	+	-	-	-	-
**F35878**	51	+	-	+	-	-	-
**F35865**	114	+	-	-	-	+	-
**F35797**	115	+	-	-	-	-	-

Supplementary Table 2. Detection of ZIKV RNA in Fetal Compartments. Indicated fetal bodily fluids were analyzed by qRT-PCR for ZIKV RNA; positive samples are denoted (+) and negative samples (-). Detection limit for assay was $1x10^4$ genomic equivalents per ml. Abbreviations: amniotic fluid (AF), cerebral spinal fluid (CSF), umbilical vein (UV), umbilical artery (UA).

	Animal ID	F35972 (♂)	F35467(♀)	F35878(♂)	F35865(♂)	F35797(♂)
	infection (dGa)	31	51	51	114	115
lymphoid	Axilliary LN	nd	nd	nd	2.8	nd
	Cervical LN	nd	nd	nd	nd	nd
	Inguinal LN	nd	nd	nd	3.7	nd
	Mesenteric LN	nd		nd	2.7	nd
	Retroperitoneal LN	nd	3.2	nd	nd	nd
	Salivary LN	nd		nd	2.9	
	Spleen	nd	nd	nd	3.4	2.7
	Thymus	3.3	3.4	nd	3.1	nd
	Tonsils	nd	3.8	nd	nd	nd
cardio-pulmonary	Heart	nd	3.1	nd	nd	nd
	Lung	nd	nd	nd	nd	nd
digestive	Gall bladder	nd		nd	nd	nd
	Liver	nd		nd	nd	nd
	Pancreas	nd		nd	nd	nd
	Parotid salivary gland	nd	3.3	nd	nd	2.5
	Submandibular gland	nd		nd	nd	nd
musculo-skeletal	Ankle	nd	nd	nd	nd	nd
	Bicep	nd	3.0	nd	3.4	2.4
	Brachioradialis	nd		nd		nd
	Elbow	nd	3.5	nd	nd	nd
	Finger	nd	3.4	nd	2.7	nd
	Hamstring	nd	3.9	nd	nd	nd
	Knee	nd	3.3	nd	nd	nd
	Quadriceps	nd	nd	nd	3.0	nd
	Skin upper torso	nd	nd	nd	nd	nd
	Soleus	nd	3.2	nd	nd	2.5
	Toe	nd		nd	nd	nd
	Tricep	nd	nd	nd	nd	nd
	Wrist	nd	nd	nd	3.7	nd
genito-urinary	Bladder	nd	3.7	nd	nd	
	Cervix		4.1			
	Epididymus	nd				
	Kidney	nd	nd	nd	nd	nd
	Ovary		3.5			
	Prostate	nd		nd	nd	nd
	Seminal vesicles	nd		nd		
	Testes	nd		nd	nd	nd
	Uterus		3.6			
neuro-endicrine	Adrenal gland	nd	nd	nd	nd	nd
	Basal Ganglion	nd		nd	nd	
	BR Cerebellum	nd	nd	nd	nd	2.4
	BR Frontal lobe		nd	nd	nd	
	BR Occipital lobe	nd	nd	nd	3.0	
	BR Parietal lobe	nd	nd	nd	nd	
	BR Stem	nd	nd	nd	nd	
	BR Temporal lobe	nd	nd	nd	2.6	
	Brachial plexus	nd	3.6	nd	3.6	nd
	Dorsal root ganglion	nd	nd	nd	nd	
	Eye	nd	4.3	nd	nd	nd
	Femoral nerve	nd		nd	nd	
	Pituitary gland	nd	3.5	nd	nd	nd
	SC Cervical	nd	3.7	nd	3.9	
	SC Lumbar	nd	3.6	nd	nd	nd
	SC Thoracic	nd	nd	nd	2.6	nd
	Sciatic nerve	nd	3.2	nd	nd	nd
	Thyroid	nd	3.3	nd	2.9	nd
	Trigeminal ganglion	nd	3.9	nd	3.0	nd

Supplementary Table 3. Viral RNA loads in fetal tissues. Indicated tissues were analyzed by qRT-PCR for ZIKV RNA. Numbers (colored backgrounds) indicate log_{10} ZIKV genomes/ µg total RNA. BR: brain; LN: lymph node; nd: not detected; blank cells indicate tissue not present or not collected.

SUPPLEMENTARY REFERENCES

1. Nyland TG, Hill DE, Hendrickx AG, Farver TB, McGahan JP, Henrickson R, et al. Ultrasonic assessment of fetal growth in the nonhuman primate (Macaca mulatta). J Clin Ultrasound. 1984;12(7):387-95. Epub 1984/09/01. PubMed PMID: 6438171.
2. Tarantal AF. Ultrasound Imaging in Rhesus (Macaca mulatta) and Long-tailes (Macaca fascicularis) Macaques: Reproductuve and Research Applications. In: S W-C, editor. The Laboratory Primate. London: Elsevier; 2005. p. 317-52.
